# Targeting the *AtCWIN1* Gene to Explore the Role of Invertases in Sucrose Transport in Roots and during *Botrytis cinerea* Infection

**DOI:** 10.3389/fpls.2016.01899

**Published:** 2016-12-20

**Authors:** Florian Veillet, Cécile Gaillard, Pierre Coutos-Thévenot, Sylvain La Camera

**Affiliations:** Laboratoire Ecologie et Biologie des Interactions, Equipe “SEVE-Sucres et Echanges Végétaux-Environnement,” UMR Centre National de la Recherche Scientifique 7267, Université de PoitiersPoitiers, France

**Keywords:** cell wall invertase, sugar transport, sucrose, *Arabidopsis thaliana*, *Botrytis cinerea*, source/sink relationship, CRISPR/Cas9

## Abstract

Cell wall invertases (CWIN) cleave sucrose into glucose and fructose in the apoplast. CWINs are key regulators of carbon partitioning and source/sink relationships during growth, development and under biotic stresses. In this report, we monitored the expression/activity of *Arabidopsis* cell wall invertases in organs behaving as source, sink, or subjected to a source/sink transition after infection with the necrotrophic fungus *Botrytis cinerea*. We showed that organs with different source/sink status displayed differential CWIN activities, depending on carbohydrate needs or availabilities in the surrounding environment, through a transcriptional and posttranslational regulation. Loss-of-function mutation of the *Arabidopsis* cell wall invertase 1 gene, *AtCWIN1*, showed that the corresponding protein was the main contributor to the apoplastic sucrose cleaving activity in both leaves and roots. The CWIN-deficient mutant *cwin1-1* exhibited a reduced capacity to actively take up external sucrose in roots, indicating that this process is mainly dependent on the sucrolytic activity of *AtCWIN1*. Using T-DNA and CRISPR/Cas9 mutants impaired in hexose transport, we demonstrated that external sucrose is actively absorbed in the form of hexoses by a sugar/H^+^ symport system involving the coordinated activity of AtCWIN1 with several Sugar Transporter Proteins (STP) of the plasma membrane, i.e., STP1 and STP13. Part of external sucrose was imported without apoplastic cleavage into *cwin1-1* seedling roots, highlighting an alternative *AtCWIN1*-independent pathway for the assimilation of external sucrose. Accordingly, we showed that several genes encoding sucrose transporters of the plasma membrane were expressed. We also detected transcript accumulation of vacuolar invertase (VIN)-encoding genes and high VIN activities. Upon infection, *AtCWIN1* was responsible for all the *Botrytis*-induced apoplastic invertase activity. We detected a transcriptional activation of several *AtSUC* and *AtVIN* genes accompanied with an enhanced vacuolar invertase activity, suggesting that the *AtCWIN1*-independent pathway is efficient upon infection. In absence of *AtCWIN1*, we postulate that intracellular sucrose hydrolysis is sufficient to provide intracellular hexoses to maintain sugar homeostasis in host cells and to fuel plant defenses. Finally, we demonstrated that *Botrytis cinerea* possesses its own functional sucrolytic machinery and hexose uptake system, and does not rely on the host apoplastic invertases.

## Introduction

Higher plants have the ability to use atmospheric CO_2_ and solar energy to produce organic compounds, mainly in the form of sugar. As sugar production only takes place in green tissues, sugar partitioning occurs between photosynthetically active leaves that produce excess of photoassimilates and heterotrophic sink organs that are dependent on the continuous supply of sugars by the sources (Ainsworth and Bush, 2011). In most plants, carbon assimilated in sources is translocated throughout the plant *via* the phloem complex as a form of sucrose to provide carbon, and energy for growth and synthesis of storage (Lemoine et al., 2013). As many different sinks compete for carbohydrate at the same time, sucrose partitioning is regulated according to the individual sink strength which is defined as the ability of heterotrophic organs to import, process, and store photoassimilates (Herbers and Sonnewald, 1998; Ainsworth and Bush, 2011).

At the cellular level, sucrose is a key carbon source for many physiological processes, i.e., growth, development, and defense, and its role as signaling molecule is well established (Koch, 2004; Rolland et al., 2006; Ruan, 2014). The metabolism of sucrose produces hexoses, which provide carbon skeletons for primary metabolism (Koch, 2004; Bolouri-Moghaddam et al., 2010). In higher plants, two classes of enzymes catalyze the cleavage of sucrose. Sucrose synthases (EC 2.4.1.13) need UDP to reversibly cleave sucrose into UDP-glucose and fructose. By contrast, invertases (EC 3.2.1.26) irreversibly catalyze sucrose cleavage into glucose and fructose (Roitsch and González, 2004). Depending on their pH optimums and subcellular localizations, invertases have been classified into three classes (Ruan, 2014). Cell wall invertases (CWIN) are enzymes ionically bound to the cell wall with an acidic optimum pH of 3.5–5 (Roitsch and González, 2004). Vacuolar (VIN) and cytoplasmic invertases (CIN) are soluble enzymes with an acidic (pH 5.0–5.5) and neutral (pH 6.8–8.0) optimum pH, respectively (Roitsch and González, 2004). Plant invertases are encoded by small gene families with specific temporal and spatial expression patterns (Ruan, 2014). They are tightly regulated from transcriptional to posttranslational levels, particularly in response to environmental stimuli (Ruan, 2014). CWINs and VINs are subjected to posttranslational control by small proteins (15–23 kDa) that have been characterized as specific inhibitors of β-fructosidases (INH) (Link et al., 2004; Rausch and Greiner, 2004; Hothorn et al., 2010; Su et al., 2016).

A wide range of regulatory roles has been proposed for cell wall invertases (Roitsch and González, 2004). By degrading sucrose in sinks, cell wall invertases participate to the osmotic gradient between source and sink that drives the long-distance transport of assimilates from source leaves into sink organs (Lemoine et al., 2013). Therefore, the function of cell wall invertases is essential for the regulation of phloem unloading and sink strength establishment.

Several studies have pointed out the crucial role played by some invertases and INHs in growth and development. Altered CWIN activity severely impacts reproductive organs that are often symplasticaly isolated from the surrounding environment. For example, mutations in *ZmCWIN2, OsCWIN2* (*OsGIF1*), and *SlCWIN1* (*Lin5*) lead to the production of miniature seeds, a decrease in grain yield and an altered flower and fruit morphology, respectively (Cheng et al., 1996; Wang et al., 2008; Zanor et al., 2009). In *Arabidopsis, atcwin4* mutant flowers did not produce nectar but accumulated more starch and less soluble sugars (Ruhlmann et al., 2010). CWIN activity also has a strong impact on the normal plant development. Antisense repression of *DcCWIN1* or *DcVIN1* genes in carrot leads to impaired growth and development with elevated levels of sucrose and starch in aerial tissues and reduced carbohydrate content in roots (Tang et al., 1999). *VIN* genes are known to play a major role in generating hexoses in fruits. For example, impaired expression of *SlVIN1* in tomato leads to 30% smaller fruits with higher sucrose content (Klann et al., 1996). Using a QTL approach, Sergeeva et al. (2006) identified *AtVIN2* as a regulator of root elongation. Unlike CWINs and VINs, the role of CINs in plant physiology is not well understood, although some studies have pointed out their role in root growth in *Arabidopsis*, rice and *Lotus japonicus* (Jia et al., 2008; Barratt et al., 2009; Welham et al., 2009).

On the other hand, numbers of studies have described the induction of CWIN expression and/or activity in response to various pathogens (Proels and Hückelhoven, 2014; Tauzin and Giardina, 2014). Pathogen-induced CWIN activity generates important modifications in plant carbon partitioning, i.e., reduction of the photosynthesis, hexose accumulation in the apoplast, then limiting long distance sucrose export, and creation of an additional sink competing with other sinks (Scharte et al., 2005; Biemelt and Sonnewald, 2006; Berger et al., 2007; Schultz et al., 2013). Differential level of CWIN activity may affect plant resistance. In rice, constitutive overexpression of the cell wall invertase gene *GRAIN INCOMPLETE FILLING 1* enhances resistance against both the bacteria *Xanthomonas oryzae* pv. *Oryzae* and the fungus *Magnaporthe oryzae* (Sun et al., 2014). In tobacco, RNA interference of CWIN genes *LIN6* and *LIN8* results in an increased susceptibility to the oomycete *Phytophtora nicotianae* (Essmann et al., 2008a). By contrast, the overexpression of INHs in *Arabidopsis* roots, resulting in the reduction of CWIN activity, leads to reduced symptoms of clubroot disease caused by the obligate biotrophic protist *Plasmodiophora brassicae* (Siemens et al., 2011).

Contrasting impacts of CWINs on plant resistance indicate that the additional pool of free hexoses generated by the activity of apoplastic invertases may be beneficial for both the pathogen and the host. For the former, these sugars represent a source of energy allowing growth and reproduction whereas for the latter, sugars can fuel the costly induction of defense mechanisms and act as signaling molecules to regulate defense gene expression (Herbers et al., 1996; Roitsch, 1999; Bolton, 2009; Bolouri Moghaddam and Van den Ende, 2012). The competition for available sugars is crucial for the outcome of the interaction (Lemoine et al., 2013). Depending on their mode of colonization, pathogens use different and complex strategies to favor infection. For example, the hemibiotrophic bacteria *X. campestris* pv. *vesicatoria* delivers type III effector XopB to interfere with CWIN activity and suppress sugar-induced defense responses in pepper leaves (Sonnewald et al., 2012). Obligate pathogens grow in the apoplast of living tissues and divert host metabolism by complex strategies of suppression or avoidance of plant defenses. Biotrophs mainly rely on the sugars transferred from the host *via* specialized feeding structures (Hall and Williams, 2000; Panstruga, 2003; Sutton et al., 2007). By contrast, necrotrophs kill host cells using an arsenal of secreted virulence factors, including toxins (van Kan, 2006; Laluk and Mengiste, 2010). They feed on dead tissues by macerating host organic polymers using cell wall degrading enzymes (Kubicek et al., 2014).

Free hexoses released by the activity of CWINs are retrieved either by host or pathogen cells through the activity of plasma membrane sugar transporters. Inductions of hexose transporter expression and/or activity have been reported in several plant-pathogen interactions (Azevedo et al., 2006; Sutton et al., 2007; Lemonnier et al., 2014). In response to the biotrophic fungus *Erysiphe cichoracearum*, the increased CWIN activity is associated with the coordinated induction of the cell wall invertase gene *AtCWIN1* and the Sugar Transporter Protein (STP) gene *STP4* (Fotopoulos et al., 2003). The same co-regulation of the Hexose Transporter *VvHT5* and a cell wall invertase has been observed in grapevine infected with biotrophic fungi (Hayes et al., 2010). In response to the necrotrophic fungus *B. cinerea*, the induction of the *Arabidopsis* hexose transporter STP13 is required for the plant basal resistance (Lemonnier et al., 2014). Conversely, sugar transporters of the SWEET family that function as facilitators of sugar efflux can be targeted by pathogens to acquire sugars for their own growth (Chen et al., 2010; Chong et al., 2014; Chandran, 2015).

To gain access to carbohydrates from the host, pathogens also possess their own cell wall invertases and hexose transporters, allowing them to compete for resources (Doidy et al., 2012). However, for most of the pathosystems, it has not been possible to discriminate the contribution of plant or fungus to the increased invertase activity (Voegele and Mendgen, 2011). Voegele et al. (2006) identified the invertase Uf-INV1 from the rust fungus *Uromyces fabae* and Ruiz and Ruffner (2002) detected a fungal invertase in grape infected with *B. cinerea*. Several pathogen sugar transporters have also been identified. The hexose transporter UfHXT1 from *U. fabae* has been described to preferentially transport glucose with high affinity (Voegele et al., 2001), while hexose transporters of the hemibiotrophic pathogen *Colletotrichum graminicola* (CgHXT1-5) displayed different enzymatic properties (Lingner et al., 2011). *Botrytis cinerea* possesses a multigenic hexose uptake system, including the fructose H^+^/symporter named *BcFRT1* (Doehlemann et al., 2005) and a family of 17 putative hexose transporters, named *BcHXTs* (Dulermo et al., 2009).

In this study, we examined cell wall invertase activities in *A. thaliana* organs exhibiting different source/sink status, and in leaves infected by the necrotrophic fungus *B. cinerea*. The expression of cell wall invertase and invertase inhibitor-encoding genes has been analyzed in normal conditions and upon infection. The insertional mutation of the *Arabidopsis* cell wall invertase 1 gene, *AtCWIN1*, revealed that the corresponding protein *AtCWIN1* was the major contributor to the CWIN activity in both leaves and roots, and was responsible for all the *Botrytis*-induced CWIN activity. We used the CWIN-deficient mutant *cwin1-1* as a tool to explore the role of cell wall invertases in the root assimilation of external sucrose and in the mechanism of apoplastic sucrose retrieval by the host during leaf infection. We proposed a model involving two different pathways for apoplastic sucrose absorption into plant cells. The major pathway depends on the *AtCWIN1*-mediated sucrose degradation and the coordinate activity of hexose-specific transporters, while an alternative pathway likely involves sucrose transporters and intracellular sucrose cleavage. The importance of both pathways for normal growth and resistance to pathogen is discussed. We further explored the assimilation of sugars by *B. cinerea*, demonstrating that the acquisition of host hexoses occurs *via* functional sucrolytic machinery and a multigenic hexose uptake system.

## Materials and methods

### Plant materials and growth conditions

The wild type *Arabidopsis thaliana* was ecotype Columbia (Col-0). For soil culture of *Arabidopsis* plants, seeds were sown in an autoclaved mix of compost/vermiculite (3/1) and placed into a growth chamber under a 10 h light (22°C)/14 h dark (18°C) photoperiod, with a 65% relative humidity. Experiments were performed on 5–6 week-old plants. Fully expanded leaves were used for sampling. Roots were separated from soil by multiple careful washings with water before sampling. For *in vitro* culture, seeds were sterilized and sown on half-strength Murashige and Skoog (MS) plates containing 0.8% agar and supplemented or not with 2% sucrose. Plates were kept in the dark for 2 days at 4°C and then placed at 22°C under a 16/8 h light/dark photoperiod. Roots from 9-day-old seedlings were sampled.

The *stp13-2* (salk_021204) and *cwin1-1* (salk_091455) mutant lines were obtained from the SALK T-DNA insertion mutant collection (Alonso et al., 2003). Plants homozygous for the mutation were identified by PCR. The *stp13-2 and STP13OE-6* mutant lines were previously described in Lemonnier et al. (2014) and *dde2-2*, defective in the allene oxide synthase gene, was characterized in von Malek et al. (2002).

### Cloning procedures

Complete coding sequence of *AtSTP1* was obtained after a PCR-amplification of cDNA made from *Arabidopsis* leaves (Phusion Taq Polymerase, Thermo Scientific). Primers for amplification were listed in Supplementary Table 1. PCR products were cloned into the pENTR-D-TOPO vector (Invitrogen), sequenced and LR-recombined with pB2GW7 destination vector (Karimi et al., 2002) to express *AtSTP1* under the control of the *CaMV35S* promoter. This construct was transferred into *Agrobacterium tumefaciens* strain GV3101 (pMp90) by heat shock. Wild type Col-0 plants were transformed using the floral dipping method (Clough and Bent, 1998) and transgenic homozygous T3 lines were selected according to their Basta resistance.

### Generation of genetically modified *Arabidopsis* plants using the CRISPR/Cas9 genome editing system

The *AtSTP1* gene was targeted using the CRISPR/Cas9 system (Jinek et al., 2012) using a protocol adapted from Fauser et al. (2014). CRISPR PLANT (Xie et al., 2014) and RGEN TOOLS (Park et al., 2015) softwares were used to design a 20 bp spacer sequence complementary to a region located in the third exon of *AtSTP1* gene (Supplementary Figure 1A, Supplementary Table 1). This sequence was inserted into the *BbsI* restriction site of the pEn-Chimera plasmid (Fauser et al., 2014). The customized sgRNA cassette was then transferred into the pDe-Cas9 vector (Fauser et al., 2014) using a single site Gateway LR reaction. The resulting plasmid was transferred into *A. tumefaciens* GV3101 (pMp90) strain by heat shock for subsequent floral dipping transformation in Col-0 plants (Clough and Bent, 1998). Primary transformants (T1) were selected according to their Basta resistance.

To genotype *AtSTP1* lines, genomic DNA was extracted from 100 T2 plants coming from 2 independent T1 lines. The detection of the T-DNA was performed by PCR using primers located on the *Cas9* gene (Supplementary Table 1). 26 T-DNA-free plants were selected and tested for the presence of mutations in the target locus by High Resolution Melting-curve (HRM) analysis (data not shown). Three plants exhibiting divergent melting-curves from the wild type were regarded as edited, and mutations were confirmed *via* Sanger sequencing (BigDye Terminator v3.1, Applied Biosystems). Mendelian inheritance of mutations and lack of the T-DNA were confirmed in T3 generation by sequencing and basta resistance (Supplementary Figure 1B). A single line showing a homozygous mutation in the *AtSTP1* gene was selected and named *CR-stp1*. The genome edition resulted in an insertion of a single nucleotide, leading to a frameshift in the coding sequence (Supplementary Figure 1B). We identified 5 loci that harbored some similarities with the target locus using RGEN TOOLS (Bae et al., 2014) and off-target potential was assessed by HRM (Supplementary Figures 1D,E). Primers used are listed in Supplementary Table 1.

### Culture of *B. cinerea*, infection methods, and fungal quantification

*B. cinerea* strain B05.10 (Staats and van Kan, 2012) was grown on Difco potato dextrose agar (Becton-Dickinson) at 22°C under a 16/8 h light/dark photoperiod. Conidia were harvested in sterile water, filtered through miracloth (EMD Chemicals) and diluted to the appropriate concentration in quarter-strength potato dextrose broth (PDB; Becton–Dickinson).

For sugar uptake assays, liquid culture of *B. cinerea* was performed by cultivating 10^4^ conidia ml^−1^ in sterile 6-well culture plates (Nunclon Delta Surface, Thermo Scientific) containing 3 ml of Gamborg B5 medium (pH 5.8) supplemented with 2% sucrose. Plates were placed at 22°C under a 16 h/8 h light/dark photoperiod with orbital shaking for 24 h. For disease assays, plants were drop-inoculated on source leaves with 6 μl of the conidia suspension (5 × 10^4^ conidia ml^−1^) and the diameter of resulting lesions was measured after 3 days. For gene expression analysis and enzymatic activities, the entire rosette was sprayed with the conidia suspension (5 × 10^4^ conidia ml^−1^) or, as a control, with quarter-strength potato dextrose broth without conidia. For fungal growth measurement, 6 μl of the conidia suspension (5 × 10^4^ conidia ml^−1^) were spotted on mature leaves. Ten 8 mm diameter disks from 3 plants were harvested at the indicated time points. DNA extraction and PCR analysis of *iASK* and *cutA* genes were performed as described by Gachon and Saindrenan (2004). After inoculation, plants were placed in a tray closed under saturating humidity and softened light. Infected source leaves were harvested at the indicated time points and frozen in liquid nitrogen.

### DNA extraction and HRM analysis

DNA from *A. thaliana* plants was extracted using the Smart Extract kit (Eurogentec) or the NucleoSpin Plant II (Macherey-Nagel) according to manufacturer instructions. HRM analysis was performed using the LightCycler® 480 II system, the High Resolution Melting Master and the LightCycler® 480 Gene Scanning Software (Roche Life science).

### RNA extraction and real-time quantitative PCR (RT-qPCR) analysis

Total RNA was extracted from frozen ground *A. thaliana* materials using TRIzol reagent (Invitrogen) according to the manufacturer instructions. For soil-grown root samples, extraction was performed as described by Kay et al. (1987). RNA quantity and quality were verified using a Multiskan GO plate reader (Thermo Fischer) and an agarose gel. Total RNA was treated with DNAse I (Sigma-Aldrich) and reverse transcription was performed using M-MLV reverse transcriptase (Promega). Real-time quantitative PCR was carried out using the GoTaq qPCR Master Mix (Promega) with a Mastercycler *realplex*^2^ instrument (Eppendorf). For *Arabidopsis* target gene expression, results were normalized with the average of the Ct value of two reference genes, *At4g26410* and *AtACTIN2* (*At3g18780*), previously described as stable genes (Czechowski et al., 2005; Lemonnier et al., 2014). For *B. cinerea* target gene expression, results were normalized to the expression of the *BcTUBA* gene (*Bc1g05600*) (Dulermo et al., 2009). The results were expressed as relative gene expression according to the 2^−ΔCt^ method described by Schmittgen and Livak (2008). Primers have been designed using Primer3 (Untergasser et al., 2012) in conjunction with Netprimer (www.premierbiosoft.com/netprimer) and tested for their specificity and efficiency (≥90%). Sequences of the primers used in this study are listed in Supplementary Table 2.

### Determination of invertase activities

Total extracts were made by mixing ground samples with 700 μl of ice-cold extraction buffer (50 mM HEPES, 1 mM EDTA, 5 mM DTT, and 1 mM PMSF). Soluble and insoluble fractions were separated by centrifugation at 20,000 g for 15 min at 4°C. The supernatant was used for cytoplasmic (CIN) and vacuolar (VIN) invertase activities measurements. The pellet was washed three times and then resuspended in 500 μl of the extraction buffer for the CWIN activity determination. Sucrolytic activities were assayed by adding samples in the incubation buffer containing 100 mM sucrose and 100 mM sodium acetate buffered at pH 4 for apoplastic activity and pH 5 for vacuolar activity. For CIN activity, samples were mixed with 100 mM sucrose and 25 mM HEPES (pH 7). The mixture was incubated at 30°C for 60 or 90 min and stopped by the addition of the stop solution (1 M sodium potassium tartrate, 1% 3,5-dinitrosalicylic acid and 0.5 M KOH). Samples were incubated at 95°C for 10 min and cooled to room temperature before absorbance measurements at 560 nm using a microplate reader (Multiskan GO, Thermo Fischer). Results were normalized to the fresh weight.

### Radiolabeled sugar uptake in *Arabidopsis* seedlings and *Botrytis* mycelium

Five entire seedlings (6-day-old) or seedling roots (9-day-old) were placed for 45 min in Petri dishes containing the equilibration buffer (20 mM MES-KOH pH 5.8, 1 mM CaCl_2_) under agitation. After equilibration, samples were transferred into the incubation buffer (20 mM MES pH 5.8, 1 mM CaCl_2_, 0.2 mM glucose or sucrose) containing radiolabeled sugars (0.1 μCi. ml^−1^ of [^14^C]-glucose or [^14^C]-sucrose) for 45 min under agitation.

A liquid culture of *Botrytis* mycelium was washed twice and incubated for 45 min in equilibration buffer (Gamborg B5, 20 mM MES-KOH pH 5.8) under agitation. Incubation buffer containing unlabeled sucrose (0.2 mM) and [^14^C]-sucrose (0.1 μCi. ml^−1^) was added to the mixture and incubated for 30 min under agitation.

After incubation, samples were washed three times for 2 min in equilibration buffer. Samples were left overnight in the digestion buffer (36.4% perchloric acid w/v, 0.017% triton X-100 w/v and 8.1% hydrogen peroxide w/v) at 60°C. Incorporated radioactivity was determined by liquid scintillation counting (Tri-Carb 2910 PR, PerkinElmer). To measure CCCP-insensitive sugar uptake, CCCP (20 μM) was added into the equilibration buffer 10 min before addition of incubation buffer. For competition experiments, competitive unlabelled sugars (4 mM for sucrose and mannitol, 2 mM each for glucose and fructose in 1:1 mixture) were added in 20-fold excess together with radiolabeled sugars in the incubation buffer.

### Soluble sugar analysis

Frozen ground *A. thaliana* materials were serially extracted (three times) in methanol/chloroform/water (60/25/15, v/v/v). The mixture was centrifuged at 1800 g for leaf extracts or 5000 g for root extracts for 10 min at 20°C. Supernatants were pooled and mixed with 1.8 ml of water and centrifuged at 1200 g for 15 min at 20°C. The supernatant was collected and evaporated in a centrifugal vacuum evaporator (MiVac QUATTRO concentrator) at 50°C for 3 h. The soluble glucose, fructose and sucrose contents of sample extracts were measured using the Sucrose/D-Fructose/D-Glucose Assay Kit (Megazyme) according to the manufacturer instructions.

### Statistical analyses

Statistical analyses were performed using the GraphPad Prism version 7.00 for Mac, GraphPad Software, La Jolla California USA, www.graphpad.com.

## Results

### Cell wall invertase activity and gene expression in *A. thaliana* organs with different source/sink status

**Figure 1:**
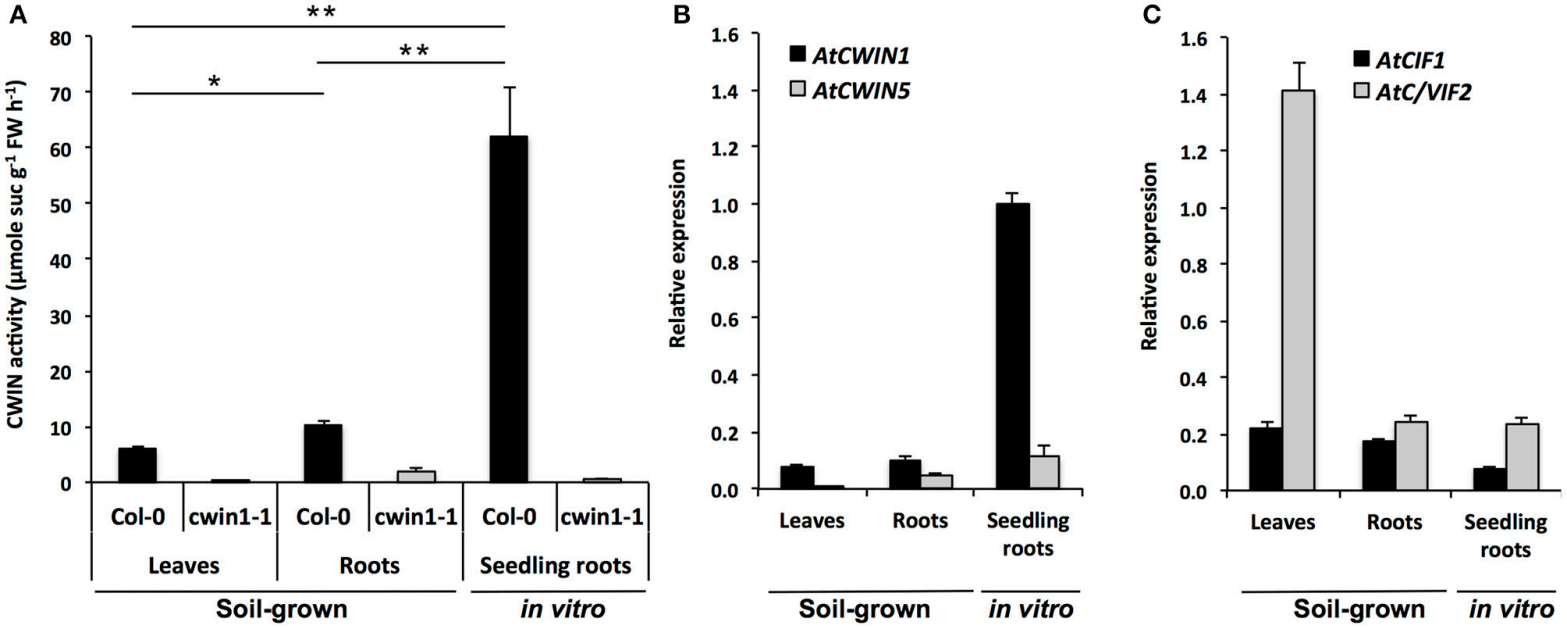
**Cell wall invertase activity and gene expression in different *Arabidopsis* organs. (A)** CWIN activity in Col-0 and *cwin1-1* was assayed from frozen ground tissues of 5–6 week-old soil-grown plants (leaves and roots) or 9-day-old seedling roots. Data represent mean (±SE) of 2 (seedling roots) or 3 (leaves and roots) independent experiments. **(B,C)** Relative gene expression of *AtCWIN1, AtCWIN5* **(B)**, *AtCIF1 and AtC/VIF2* **(C)** genes was performed by RT-qPCR and results were normalized to the plant reference genes *At4g26410* and *AtACTIN2* (*At3g18780*). Data represent mean (±SE) of 3 independent experiments. *AtCWIN2 and -4* are not presented because transcript levels were below the detection threshold. For **(A)**, asterisks represent significant differences between organs of Col-0 plants (Student's *t*-test, ^*^P < 0.05; ^**^P < 0.01).

Cell wall invertases (CWINs), that mediate the sucrose cleavage into hexoses in the apoplast, are key players in plant carbon partitioning between source and sink organs. Our first aim was to quantify the level of CWIN activity in different organs of *Arabidopsis* plants and to identify *AtCWIN* gene(s) that could be responsible for these CWIN activities. Insoluble fractions of protein extracts were assayed for CWIN activity in mature leaves and roots from adult soil-grown plants, and in roots grown *in vitro* (Figure 1A). These organs were chosen because they are indicative for different source/sink status. Plants grown in soil are representative of an autotrophic situation, in which typical source/sink relationship occurs with source leaves supplying photoassimilates to carbon sinks (i.e., roots). In heterotrophic situation as *in vitro* culture, roots can retrieve carbohydrates directly from the medium and export them to other parts of the plant. In this case, one can suppose that the source/sink balance is modified compared to the autotrophic situation (Wolf et al., 1998).

Roots of soil-grown *A. thaliana* displayed CWIN activity that was significantly 2-fold higher than in leaves (Figure 1A). The higher CWIN activity in roots is consistent with a role of CWIN in the creation of a gradient of sucrose between source and sink. Strikingly, in the presence of non-limited sucrose availability in the culture medium, the level of CWIN activity in roots grown *in vitro* was 6-fold higher than the one of soil-grown roots (Figure 1A). To identify genes that could contribute to these CWIN activities, we monitored the expression profile of genes encoding functional CWINs (Figure 1B). In *Arabidopsi*s genome, among the 6 annotated *AtCWIN* genes, only *AtCWIN1*, -*2*, -*4*, and -*5* encode functional proteins with CWIN activity, since *AtCWIN3* and -*6* were found to be fructan exohydrolases (FEH) (De Coninck et al., 2005). *AtCWIN2* and -*4* transcripts were not detected in leaves and roots, which is in agreement with a previous study showing their specific expression in reproductive tissues (Wang and Ruan, 2012). In source leaves, *AtCWIN1* was the most expressed *AtCWIN* gene whereas *AtCWIN5* transcripts were barely detected. Both genes were expressed in soil-grown roots at a detectable level (Figure 1B). In concordance with data from enzymatic activity, we found that the expression of *AtCWIN1* in roots cultured *in vitro* was approximately 10-fold higher compared to roots grown in soil (Figure 1B).

To address a potential posttranslational regulation, we monitored the expression of *AtCIF1* and *AtC/VIF2* genes encoding specific invertase inhibitor proteins (INHs) (Link et al., 2004; Su et al., 2016). In source leaves, we observed a high accumulation of *AtC/VIF2* transcripts whereas in roots, both genes were expressed at similar low levels (Figure 1C).

Taken together, these data showed a strong correlation between *AtCWIN1* expression profile and CWIN activities in the different organs we tested. This will contribute to the assumption that CWIN activity is transcriptionally regulated, with a major role of *AtCWIN1*. In source leaves, the low level of CWIN activity is probably maintained by the posttranslational activity of invertase inhibitors, such as AtC/VIF2. Collectively, it indicates that organs with different source/sink status displayed differential CWIN activities, which can be correlated with carbohydrate needs or availabilities in the surrounding environment.

### *AtCWIN1* is essential for cell wall invertase activities in *A. thaliana* roots and leaves

To investigate the contribution of the *AtCWIN1* protein to the overall CWIN activity in leaves and roots, we isolated a homozygous T-DNA mutant (*cwin1-1*), impaired in *AtCWIN1* expression (Supplementary Figure 2). As seen in Figure 1A, CWIN activity was completely abolished in *cwin1-1* source leaves and dramatically reduced in roots grown in soil and cultured *in vitro*. These results demonstrate that *AtCWIN1* is responsible for all the CWIN activity in source leaves and is the major contributor to this activity in roots. The residual CWIN activity measured in *cwin1-1* roots likely involved the low participation of an additional CWIN protein, which is probably AtCWIN5 according to the transcriptional data (Figure 1B).

### *AtCWIN1*-mediated CWIN activity regulates sugar retrieval from the surrounding environment

**Figure 2:**
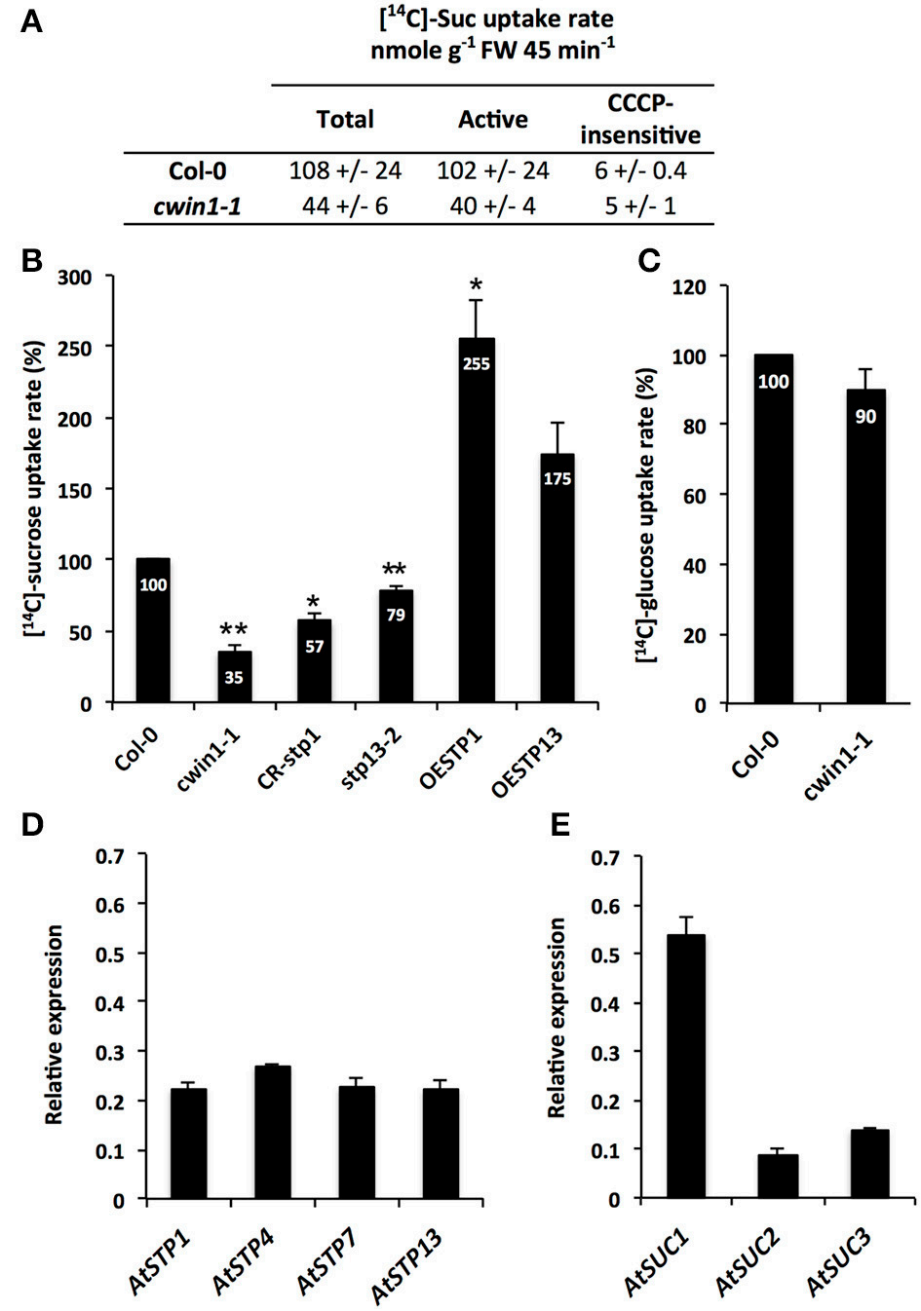
**Sucrose uptake activity and gene expression in seedling roots. (A)** [^14^C]-sucrose uptake activity in 9-day-old Col-0 and *cwin1-1* seedling roots grown with 2% sucrose (mean +/− SE of 2 independent experiments). Active sucrose uptake results from the difference between total and CCCP-insensitive uptakes **(B)** Relative [^14^C]-sucrose uptake rate in 9-day-old seedling roots of *cwin1-1, CR-stp1, stp13-2*, OESTP1, and OESTP13 lines grown with 2% sucrose. Results are relative to the Col-0 uptake rate and represent mean (±SE) of at least 3 independent experiments. **(C)** Relative [^14^C]-glucose uptake activity in 9-day-old seedling roots of Col-0 and *cwin1-1* lines. Results are relative to the Col-0 uptake rate and represent mean (±SE) of 3 independent experiments. **(D,E)** Expression of the 14 *AtSTP* **(D)** and 9 *AtSUC* **(E)** genes in 9-day-old *Arabidopsis* seedling roots. Genes that were expressed below the detection level are not presented. Gene expression analysis was performed by RT-qPCR and results were normalized to the plant reference genes *At4g26410* and *AtACTIN2* (*At3g18780*). Data represent mean (±SE) of 3 independent experiments. For **(B)** and **(C)**, asterisks represent significant differences compared to Col-0 plants (Student's *t*-test, ^*^P < 0.05; ^**^P < 0.01).

To gain insight into the regulatory role of CWIN activity in the assimilation of carbohydrates by sink organs, we further investigated the sugar absorption of 9-day-old seedling roots grown on MS medium (Figure 2). In this condition, we previously showed that CWIN activity is very high and almost totally dependent on *AtCWIN1* (Figure 1A). When grown with sucrose as the sole carbon source, Col-0 roots displayed a substantial initial rate of [^14^C]-sucrose uptake (108 ± 24 nmol g^−1^ FW 45 min^−1^) (Figure 2A). Radiolabeled sucrose retrieval by roots was mainly driven by an active sugar/H^+^ symport system since it was almost totally inhibited by the protonophore carbonylcyanide m-chlorophenyl hydrazine (CCCP) (Figure 2A). In these assays, we were not able to determine the proportion of radiolabeled sucrose that was imported as such and the one of radiolabeled hexoses formed by the cell wall invertase activity prior root absorption. To find answers, we took advantage of the availability of the *cwin1-1* mutant impaired in the apoplastic sucrose cleavage. Experiments of [^14^C]-sucrose assimilation showed that *cwin1-1* seedling roots exhibited a 65% reduction of the total [^14^C]-uptake compared to Col-0 roots (Figure 2B). Since *cwin1-1* roots exhibited a total glucose uptake rate that was very similar to Col-0 roots (Figure 2C), these results suggest that most of the external sucrose is converted into hexoses by the activity of *AtCWIN1* before absorption into Col-0 root cells. Because external sucrose was substantially absorbed in the form of hexoses, it implies the coordinated activity of hexose-specific plasma membrane carriers to transport them from the apoplast to the cytosol. Yamada et al. (2011) previously showed that hexose/H^+^ transporters, belonging to the Sugar Transporter Protein (STPs) family, play important roles in roots during the absorption of monosaccharides from the rhizosphere. AtSTP1 and AtSTP13 were major contributors to this process under normal conditions and in response to environmental stresses. Then, we monitored the expression of the 14 *AtSTP*-encoding genes in our experimental conditions. According to Yamada et al. (2011), we found that only 4 *AtSTP* genes (*AtSTP1*, -*4*, -*7*, and -*13*) were expressed to a detectable level, although they displayed similar transcript levels in our conditions (Figure 2D).

To explore the involvement of AtSTP1 and -13 in [^14^C]-sucrose retrieval, we analyzed transgenic lines with AtSTP1- or AtSTP13-mediated alterations of glucose uptake activities. We previously reported in Lemonnier et al. (2014) that the T-DNA insertion mutant *stp13-2* and the AtSTP13-overexpressing line (OESTP13) exhibited a 30% reduction or a 3-fold increase in glucose absorption compared to wild type, respectively. Here, we also generated plants constitutively expressing the complete *AtSTP1* coding sequence under the control of the CaMV35S promoter (OESTP1) and plants with a CRISPR/Cas9-mediated *AtSTP1* gene disruption (*CR-stp1*) (Supplementary Figure 1). In comparison with wild type, *CR-stp1*, and OESTP1 lines displayed a 40% decrease and a 3-fold increase of glucose uptake rates, respectively (Supplementary Figure 1C). When floated with [^14^C]-sucrose, the amount of [^14^C]-sugars that was taken up into *CR-stp1* and *stp13-2* roots was significantly reduced compared to wild type (Figure 2B). Taking into consideration that both AtSTP1 and 13 have been characterized as hexose-specific transporters with no sucrose transport activity (Sauer et al., 1990; Norholm et al., 2006; Büttner, 2007, 2010), these data strengthen our assumption that *AtCWIN1* cleaves external sucrose into hexoses before their cellular absorption by hexoses-specific transporters. Conversely, roots of either AtSTP1- and STP13-overexpressing lines showed increased [^14^C]-sugar uptake rates (Figure 2B). These data suggest that the *AtCWIN1*-mediated sucrose conversion is fast enough to supply hexoses to roots of plants exhibiting increased hexose uptake activities (OESTP1 and -13 lines).

### An *AtCWIN1*-independent pathway for root absorption of external sucrose

**Figure 3:**
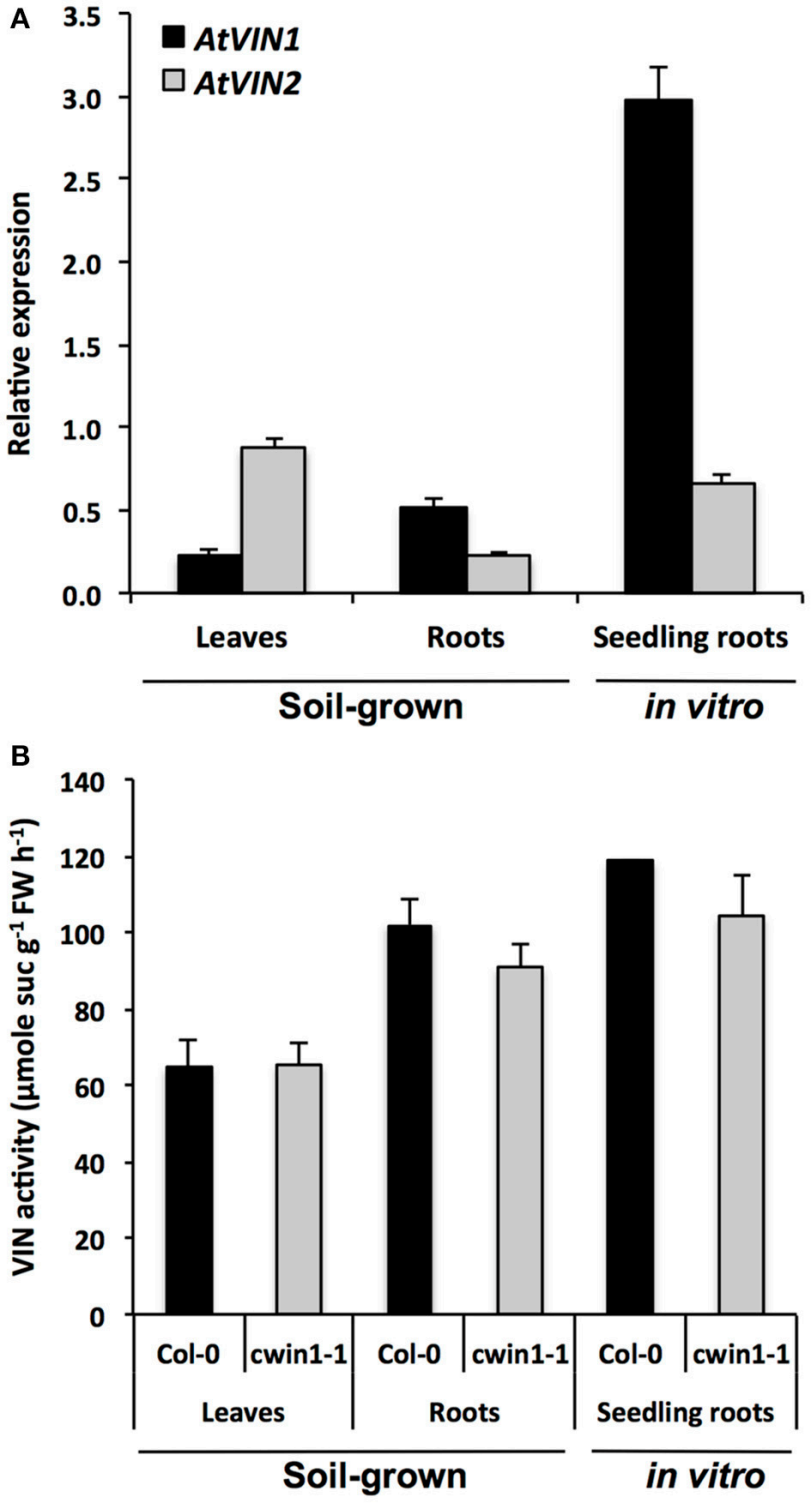
**Vacuolar invertase gene expression and activity in different *Arabidopsis* organs. (A)** Relative gene expression of *AtVIN* genes was performed by RT-qPCR and results were normalized to the plant reference genes *At4g26410* and *AtACTIN2* (*At3g18780*). Data represent mean (±SE) of 3 independent experiments. **(B)** VIN activity was assayed from frozen ground tissues of 5–6 week-old soil grown plants (leaves and roots) or 9-day-old seedling roots. Data represent mean (±SE) of 2 (seedling roots) or 3 (leaves and roots) independent experiments. No significant difference was determined between Col-0 and *cwin1-1* by a Student's *t*-test (*P* < 0.05).

Although CWIN activity was almost inexistent, the phenotypic analysis of *cwin1-1* did not reveal any obvious alteration of growth or development when cultured in soil or on MS medium (supplemented or not with 2% sucrose) (Supplementary Figures 3A,B). The soluble sugar contents in *cwin1-1* leaves and roots were also unchanged compared to wild type plants (Supplementary Figures 4A,B). This indicates that *cwin1-1* mutant is able to compensate the lack or the strong reduction of the CWIN activity either in source or sink organs. Therefore, the residual sucrose uptake activity exhibited by *cwin1-1* roots (Figure 2B) pointed out an alternative *AtCWIN1*-independent pathway for the assimilation of external sucrose. In such pathway, specific sucrose transporters should play a central role, which is supported by the detection of transcripts of 3 genes (*AtSUC1, 2*, and *3*) encoding plasma membrane sucrose carriers (AtSUCs) in roots (Figure 2E). While *AtSUC1* expression was long considered to be pollen specific (Stadler et al., 1999), recent studies revealed expression also in leaves and roots (Sivitz et al., 2007; Durand et al., 2016). These genes were recently identified as potential contributors for carbon export to the roots during water deficit (Durand et al., 2016). An *AtCWIN1*-independent pathway would also require a subsequent intracellular sucrose cleavage into hexoses for metabolic use. Since the vacuole constitutes a large reservoir for internal hexoses and sucrose (Nadwodnik and Lohaus, 2008; Hedrich et al., 2015), we further examined vacuolar invertases (AtVINs) in leaves, roots and *in vitro*-cultured roots (Figure 3). Two genes are known to encode *AtVINs* in *Arabidopsis* genome, named *AtVIN1* and *AtVIN2* (Ruan, 2014). In our conditions, both genes were expressed with differential expression patterns (Figure 3A). Interestingly, rates of sucrose cleavage by VINs were higher than the ones of CWINs in all the organs we tested (Figures 1A, 3B) and were not modified in *cwin1-1* mutant (Figure 3B). These data, together with the normal soluble sugar content in *cwin1-1* leaves and roots (Supplementary Figure 4), indicate that VIN activity may provide a substantial contribution to the internal free hexose generation and may therefore help to support the normal growth of CWIN-deficient plants.

To summarize, we demonstrated that there are two routes for the root absorption of sucrose from the surrounding environment. The major pathway is largely dependent on the sucrolytic activity of *AtCWIN1* and requires the cooperation of Sugar Transport Proteins, whereas the *AtCWIN1*-independent pathway likely involves SUC transporters and VIN activities.

### *AtCWIN1* expression is responsible for the *Botrytis*-induced CWIN activity in infected leaves

**Figure 4:**
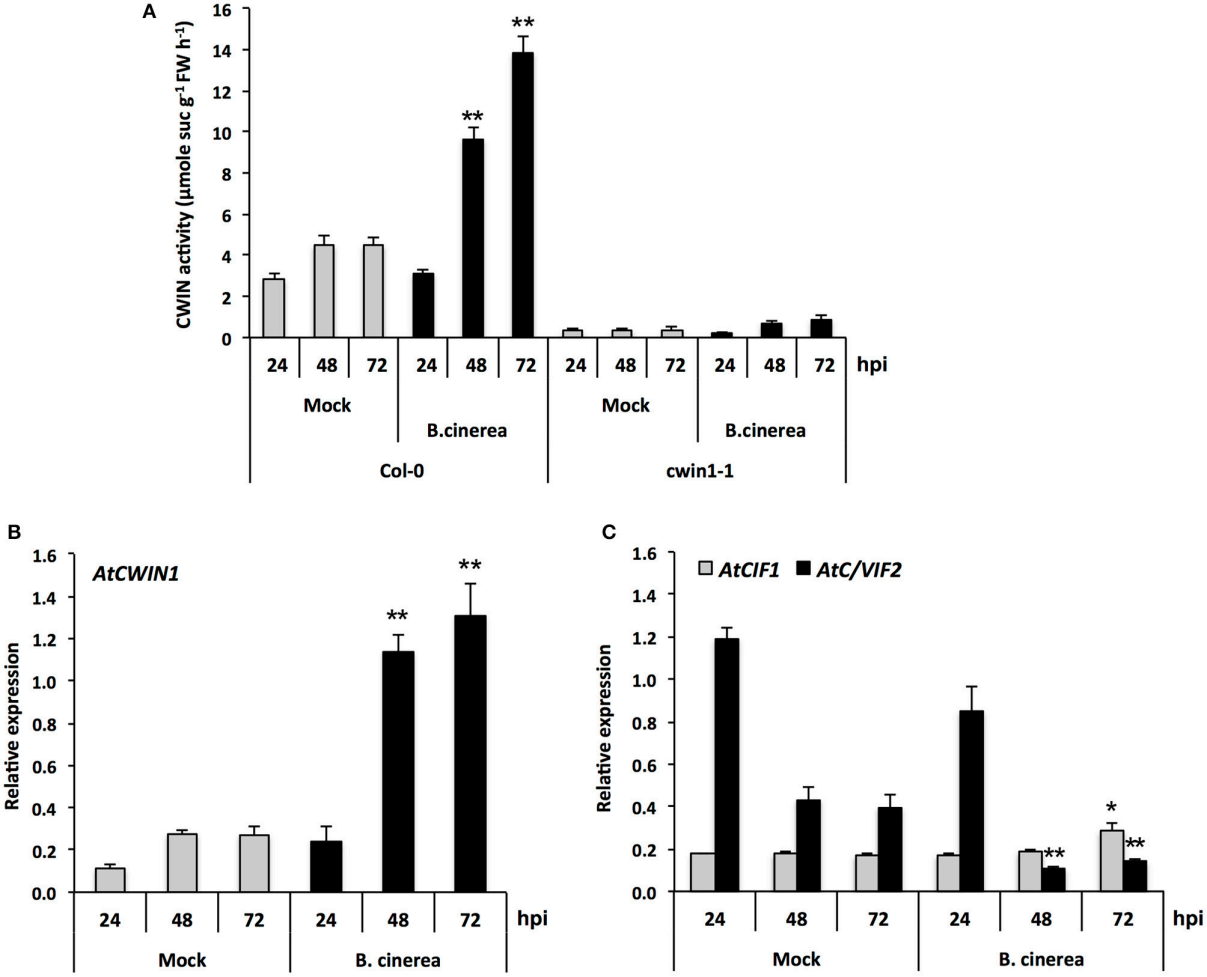
**CWIN activity and expression of *AtCWIN1*, *AtCIF1*, and *AtC/VIF2* genes in response to *B. cinerea* infection. (A)** CWIN activity in Mock and *B. cinerea* (5 × 10^4^ conidia ml^−1^) treated leaves of Col-0 and *cwin1-1* plants. Data represent mean (±SE) of at least 3 independent experiments. Relative expression of *AtCWIN1* **(B)**, *AtCIF1*, and *AtC/VIF2* **(C)** was performed by RT-qPCR in Mock and *B. cinerea* (5 × 10^4^ conidia ml^−1^) treated leaves of wild type plants. Gene expression was normalized to the plant reference genes *At4g26410* and *AtACTIN2* (*At3g18780*). Data represent mean (±SE) of at least 3 independent experiments for each condition. Asterisks represent significant differences compared to the corresponding Mock condition (Student's *t*-test, ^*^P < 0.05; ^**^P < 0.01).

CWINs have also been described as key enzymes during plant pathogen/interactions (Schultz et al., 2013; Tauzin and Giardina, 2014). While the activation of CWIN gene expression and activity has been well documented in response to several biotrophic pathogens, there is little information concerning how CWINs impact the outcome of the interaction with necrotrophic fungi, such as *B. cinerea* (Berger et al., 2007; Tauzin and Giardina, 2014). To investigate whether CWIN proteins could play a role in response to *B. cinerea*, protein extracts from infected leaves were assayed for CWIN activity. Time course experiments revealed that infection with *B. cinerea* results in a 2-fold increase of the CWIN activity from 48 h post-inoculation (hpi) compared to uninfected controls, with a 3-fold induction peak after 72 h (Figure 4A). We further monitored the expression profile of genes encoding *AtCWINs* by RT-qPCR during the course of the infection. Solely *AtCWIN1* was expressed and displayed a clear induction in leaves 48 and 72 h after infection (Figure 4B). Transcripts of the invertase inhibitor-encoding gene *AtC/VIF2* decreased dramatically to weak level from 48 hpi, while the level of *AtCIF1* expression was relatively weak and stable (Figure 4C). These results suggest that the invertase inhibitors have potentially a minor role in the control of the CWIN activity upon infection.

In *cwin1-1* mutant, inactivation of *AtCWIN1* is accompanied by a dramatic reduction of the CWIN activity in control and infected leaves (Figure 4A). In this latter case, the *Botrytis*-induced increase in CWIN activity was totally abolished (Figure 4A). As the quantity of *AtCWIN5* transcripts were detected at a negligible level (data not shown), these results stated that *AtCWIN1* was responsible for all the *Botrytis*-induced increase in CWIN activity during the course of the infection. A low residual activity was measured in *Botrytis*-infected *cwin1-1* leaf extracts (Figure 4A). Therefore, we cannot rule out a minor contribution of *Botrytis* CWINs.

In addition, we found that *AtCWIN1* transcripts accumulated concomitantly with several *Botrytis*-responsive defense genes, i.e., *AtPDF1.2, AtPR1*, and *AtPAD3* (Glazebrook, 2005) (Supplementary Figure 5) while symptoms of necrosis were visible (data not shown). Because *AtCWIN1* is expressed once the interaction between both partners is well established, it likely suggests that it may participate to the transition of healthy sources into sink, by increasing the sink strength of infected leaves.

### Deficiency in CWIN activity does not alter plant basal resistance to *B. cinerea*

**Figure 5:**
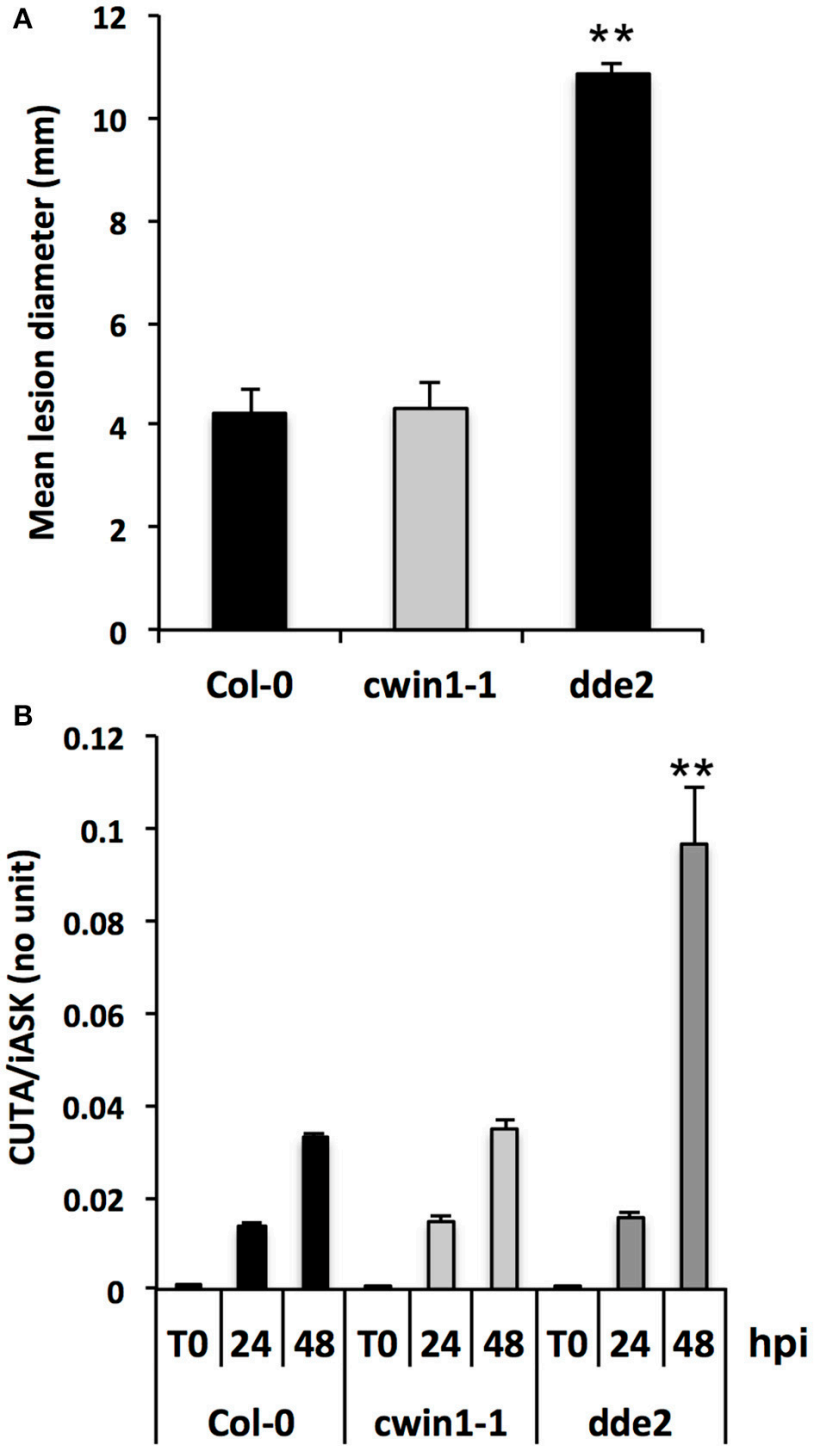
**Disease phenotype of Col-0 and *cwin1-1* plants in response to *B. cinerea* infection. (A)** Lesion diameters measured 3 days after *B. cinerea* infection. Source leaves were drop-inoculated with a PDB solution containing 5 × 10^4^ conidia ml^−1^. Data represent mean (±SE) of at least 3 independent experiments. **(B)** Quantification of *in planta* growth of *B. cinerea*. Foliar disks were cut around the infection point, and relative genomic DNA level was quantified by qPCR using *B. cinerea CUTINASE* and *Arabidopsis iASK* primers. Data represent mean (±SE) of 3 independent experiments. Asterisks represent significant differences compared to Col-0 plants (Student's *t*-test, ^**^P < 0.01).

CWIN activity has been associated with plant resistance and defense mechanisms during several biotrophic and hemibiotrophic interactions (Herbers et al., 1996; Essmann et al., 2008a; Bonfig et al., 2010; Sun et al., 2014). The absence of *Botrytis*-induced CWIN activity in *cwin1-1* (Figure 4A) prompted us to investigate the biological importance of *AtCWIN1* in the outcome of *B. cinerea* infection. To this end, *cwin1-1* plants were challenged with *B. cinerea* and tested for their susceptibility (Figure 5). Mature leaves were drop-inoculated with a PDB solution containing conidia and the average diameter of resulting lesions was used as an indicator of disease susceptibility. The jasmonic acid-defective mutant *dde2-2* was used as control for susceptible plants (von Malek et al., 2002; La Camera et al., 2011). As shown in Figure 5A, WT and *cwin1-1* plants exhibited the same susceptibility to *B. cinerea* isolate B05.10, as no significant modification of lesion size has been measured between both genotypes. In addition, the induction pattern of several *Botrytis*-responsive genes did not differ from that observed in *cwin1-1* (Supplementary Figure 5). Considering these results, we conclude that *AtCWIN1* is not involved in the supply of hexoses for activation of the PAMP-triggered Immunity. We postulated that the suppression of the sucrose-degrading activity in *cwin1-1* may alter the apoplastic sugar content at the site of infection and in turn affect the fungal development and pathogenicity. To assess this hypothesis, we monitored the fungal DNA content in leaves by real-time PCR during the course of the interaction (Gachon and Saindrenan, 2004). As seen in Figure 5B, the progression of *B. cinerea* in leaf tissues was similar in wild type and *cwin1-1* plants. These data strongly support the assumption that *AtCWIN1*-related invertase activity is not a critical step to supply hexoses for pathogen nutrition.

### Sucrose transporters expression and intracellular invertase activity are induced after infection with *B. cinerea*

**Figure 6:**
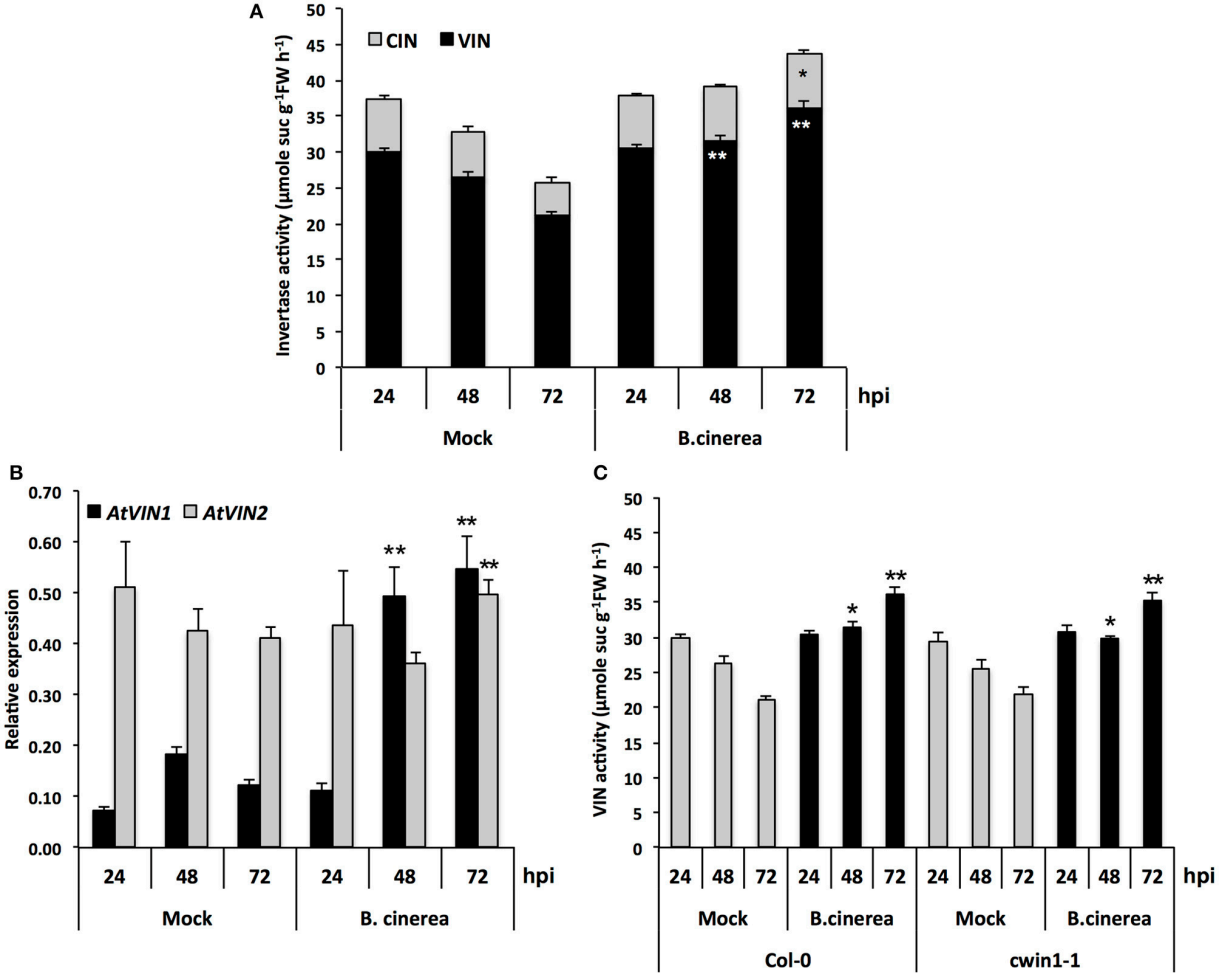
**Intracellular invertase activity and *AtVINs* gene expression analysis upon *B. cinerea* infection. (A)** VIN and CIN activities in Mock and *B. cinerea* (5 × 10^4^ conidia ml^−1^) treated leaves of Col-0 plants. Data represent mean (±SE) of at least 3 independent experiments. **(B)** Expression of *AtVIN* genes in Mock and *B. cinerea* (5 × 10^4^ conidia ml^−1^) treated leaves of Col-0 plants. Relative gene expression was performed by RT-qPCR and results were normalized to the plant reference genes *At4g26410* and *AtACTIN2* (*At3g18780*). Data represent mean at least 3 independent experiments. **(C)** VIN activity in Mock and *B. cinerea* (5 × 10^4^ conidia ml^−1^) treated leaves of Col-0 and *cwin1-1* plants. Data represent mean (±SE) of 3 independent experiments. **(A,C)** are the results of two sets of experiments. Asterisks represent significant differences compared to the corresponding Mock condition (Student's *t*-test, ^*^P < 0.05; ^**^P < 0.01).

**Figure 7:**
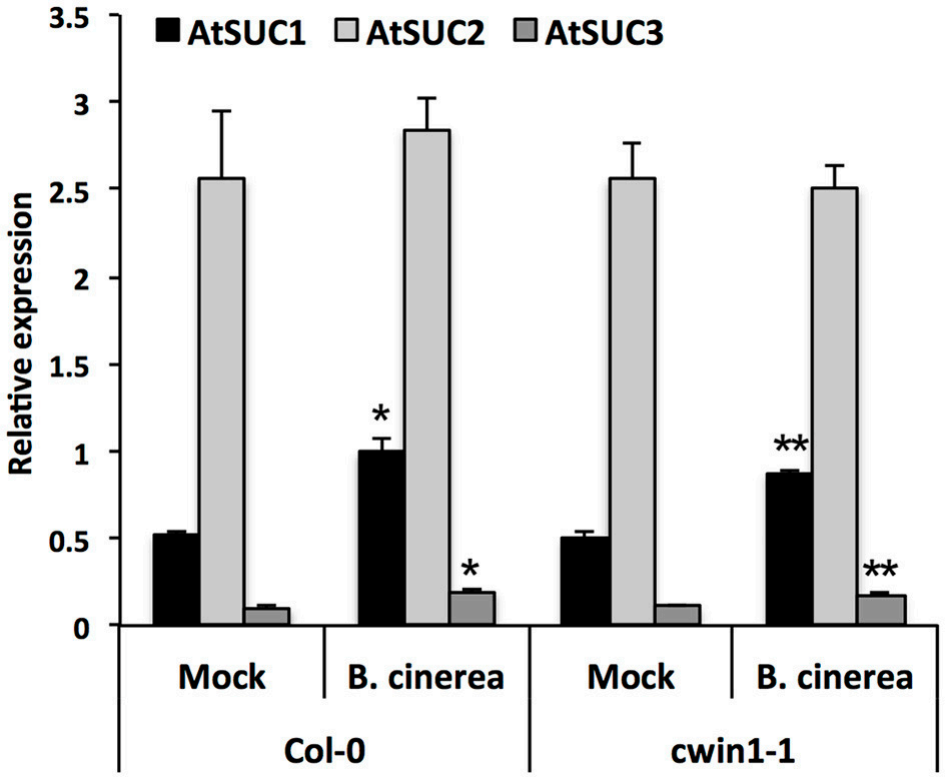
**Expression of *AtSUC* genes upon *B. cinerea* infection**. Relative gene expression of *AtSUC1*, -*2*, -*3*, -*4*, and -*5* was performed by RT-qPCR on *B. cinerea* treated leaves (5 × 10^4^ conidia ml^−1^) of wild type and *cwin1-1* plants 48 h post-inoculation. Genes that were expressed below the detection level are not presented. Results were normalized to the plant reference genes *At4g26410* and *AtACTIN2* (*At3g18780*). Data represent mean (±SE) of at least 2 independent experiments. Asterisks represent significant differences compared to the corresponding Mock condition (Student's *t*-test, ^*^P < 0.05; ^**^P < 0.01).

To provide additional clarification regarding how low level of CWIN activity in *cwin1-1* did not impact plant basal resistance to *B. cinerea*, we investigated the alternative *AtCWIN1*-independent sucrose cleavage that may occur in challenged leaves. To this end, we monitored activities of intracellular invertases, i.e., cytoplasmic (CIN) and vacuolar invertases (VIN), during the course of *B. cinerea* infection. As shown in Figure 6A, intracellular sucrolytic activities were induced at 48 and 72 h post-inoculation compared to mock treatment. VIN were approximately four times higher than the ones related to CINs. We also pointed out that VINs mainly contributed to the increased intracellular invertase activities in challenged leaves (Figure 6A). This result was supported by the transcriptional activation of genes encoding vacuolar invertases, *AtVIN1* at 48 and 72 hpi and *AtVIN2* at 72 hpi (Figure 6B). We further examined VIN activity in *cwin1-1* upon infection and found that vacuolar cleavage of sucrose was not affected, since it exhibited similar rates compared to wild type (Figure 6C). Collectively, these data strongly suggest that the high intracellular sucrolytic activity may supply free hexoses to cells undergoing PAMP-Triggered Immunity, and to this extent largely compensates the apoplastic invertase deficiency caused by the inactivation of *AtCWIN1*. This assumption likely involved the activity of plasma membrane sucrose-specific transporters to move sucrose from the apoplasm into the cytosol. Then, we monitored the expression of sucrose transporters of the plasma membrane 48 h after challenge with *B. cinerea*. The expression of *AtSUC1, 2*, and *3* were detected, *AtSUC2* transcripts being the most abundant (Figure 7). It is well established that *AtSUC2* is expressed in the collection phloem of mature leaves, and is essential in phloem loading (Truernit and Sauer, 1995; Stadler and Sauer, 1996). Interestingly, levels of *AtSUC1* and *3* transcripts were significantly increased upon *B. cinerea* infection whereas *AtSUC2* expression was not affected (Figure 7). Expression patterns of *AtSUCs* were also similar in *cwin1-1* (Figure 7). The expression of several *SUC* genes and the transcriptional up-regulation of some of them emphasized the putative role of these sucrose transporters in the cellular absorption of apoplastic sucrose into infected cells.

Collectively, these data suggest that sucrose uptake and its subsequent intracellular cleavage may constitute an efficient way to generate intracellular hexoses upon *B. cinerea* infection.

### *B. cinerea* possesses a sucrolytic machinery and a multigenic hexose uptake system for plant hexose resorption

**Figure 8:**
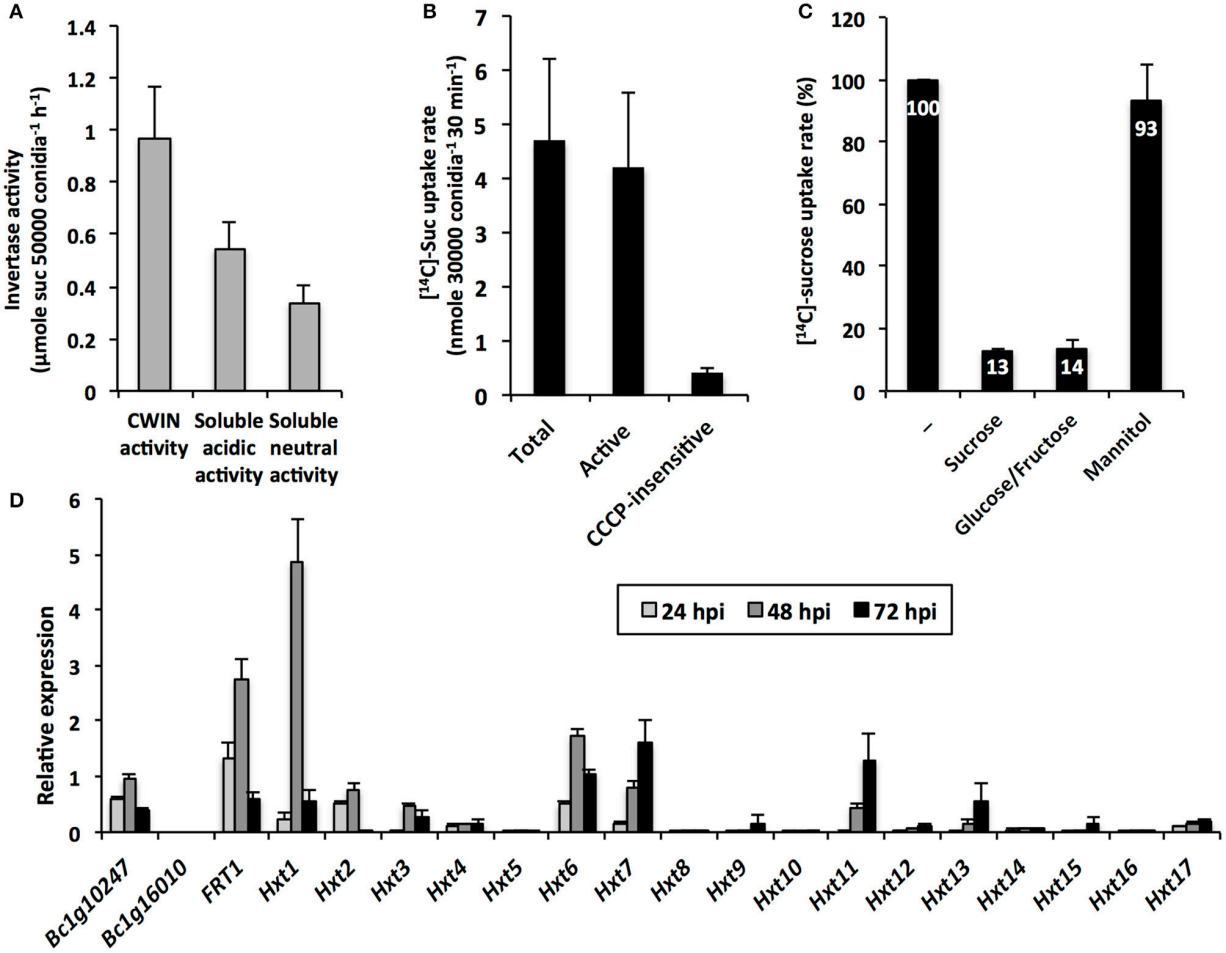
***B. cinerea*** **invertase activity, sugar transport and gene expression (A)** Cell wall and intracellular (acidic and neutral) invertase activities in *B. cinerea* mycelium grown *in vitro* (mean +/− SE of 3 independent experiments). **(B)** [^14^C]-sucrose uptake activity in *B. cinerea* mycelium grown *in vitro* (mean +/− SE of 3 independent experiments). Active sucrose uptake results from the difference between total and CCCP-insensitive uptakes. **(C)** Inhibition of [^14^C]-sucrose uptake activity by sucrose, glucose:fructose (1:1 mixture) and mannitol supplied in 20-fold excess in *B. cinerea* mycelium grown *in vitro* (mean +/− SE of at least 2 independent experiments). **(D)** Relative expression of *B. cinerea* putative invertase (*Bc1g10247* and *Bc1g16010*), fructose (BcFRT1) and hexoses transporter (*Hxt1-17*) genes in infected *Arabidopsis* leaves (5 × 10^4^ conidia ml^−1^). Gene expression analysis was performed by RT-qPCR and results were normalized to the *B. cinerea TUBA* gene. Data represent mean (±SE) of 3 independent experiments.

The cleavage of apoplastic sucrose and the likely increase of the apoplastic pool of hexoses are potentially beneficial for the heterotrophic *B. cinerea*. In the present study, we have pointed out the probable involvement of BcCWINs in protein extracts from infected leaves, as a residual CWIN activity was detected in *cwin1-1* infected plants (Figure 4A). To go further, we explored the insoluble (CWIN) and soluble (acidic and neutral) invertase activities from *B. cinerea* mycelium (Figure 8A). As it was technically unachievable to discriminate fungal material from host tissues during *in vivo* infection, protein fractions were extracted from a liquid culture of mycelium. In this condition, we were able to measure extracellular invertase activity and also to a lesser extent acidic and neutral invertase activities from the soluble protein fraction (Figure 8A). These results suggest two potential routes for apoplastic sucrose uptake. Using radiolabeled sucrose, we demonstrated that external sucrose that was taken up by fungal mycelium involved a sugar/H^+^ symport system as the protonophore CCCP inhibited almost 95% of total uptake (Figure 8B).

To investigate the CWIN dependency of the external sucrose uptake, we performed competition assays in which [^14^C]-sucrose uptake into mycelium was tested with competing sugars supplied in a 20-fold higher concentration. As seen in Figure 8C, [^14^C]-sugar uptake rates were strongly reduced by an excess of sucrose, but also by an excess of a 1: 1 mixture of glucose and fructose. As a control, we showed that the polyol mannitol did not affect fungal sucrose uptake (Figure 8C). Because the competitive effect of sucrose on [^14^C]-uptake was to the same extent as glucose/fructose (87 and 86% of inhibition, respectively, Figure 8C), we concluded that external sucrose is preferentially converted into hexoses by BcCWIN activities prior to be internalized. To date, there is no information regarding the identification of *B. cinerea* protein exhibiting CWIN activity. In our attempt to identify candidate gene(s) for such activity, we focused our interest on two genes, *Bc1g10247* and *Bc1g16010*. These genes were identified by Parrent et al. (2009) after the analysis of the evolutionary history of the glycoside hydrolase family 32 (GH32) in 76 fungal genomes. According to conserved motifs, Bc1g10247 belongs to the GH32 group 1 with a putative extracellular invertase activity, whereas Bc1g16010 is a member of the group 4 annotated as endo-inulinase (Parrent et al., 2009). We designed specific primers for both sequences and monitored their expression by RT-qPCR during the course of infection of *Arabidopsis* leaves by *B. cinerea* (Figure 8D). No transcript was found for *Bc1g16010*, which was not surprising, as *A. thaliana* does not produce fructan (Ruan, 2014). By contrast, *Bc1g10247* is expressed during the course of the interaction with *A. thaliana* (Figure 8D). As this gene is the sole annotated extracellular invertase in *B. cinerea* genome, this makes it a good candidate for this role.

Because sucrose was preferentially taken up in the form of hexoses by fungal cells (Figure 8C), we examined the expression pattern of *Botrytis* genes that were predicted to belong to the major facilitator superfamily of hexose transporters (Figure 8D). Dulermo et al. (2009) and Doehlemann et al. (2005) identified an extended gene family of hexose (17 *BcHXTs*) and fructose-specific (*BcFRT1*) transporters. As shown in Figure 8D, hexose transporter genes displayed differential expression patterns during the course the infection, with three genes that were predominantly induced at 48 hpi, i.e., *BcFRT1, BcHXT1*, -*6*, and two genes with a latter peak of expression, i.e., *BcHXT7*, and -*11*. These data are in line with the results demonstrating the capacity of *Botrytis* cells to import hexoses *in vitro* (Figure 8C). Three hexose transporter genes were also co-expressed with the putative extracellular invertase-encoding gene *Bc1g10247* (Figure 8D). As *Botrytis* is able to degrade external sucrose (Figure 8A), these data point out a strong cooperation between BcCWIN(s) and a multigenic hexose uptake system for the retrieval of host sugars and provide strong support to the assumption that infection by pathogens creates a new sink.

## Discussion

### Role of CWIN activity in carbon partitioning

Because cell wall invertases are described as key regulators of the plant carbon partitioning and the sink strength, our first goal was to explore and compare the role of *Arabidopsis* cell wall invertases in organs behaving as source, sink or subjected to a source/sink transition. We found that CWIN activity was higher in roots than in leaves (Figure 1A), which is in agreement with the proposed role of root apoplastic invertases, thought to facilitate phloem unloading by maintaining a sucrose concentration gradient between source and sink organs (Ruan, 2014). Plants grown *in vitro* with an exogenous source of carbon are believed to be heterotrophic. In cultured potato plants, the analysis of CO_2_ assimilation showed that high sugar concentration in medium reduced the contribution of carbon derived from the photosynthesis to the plant growth (Wolf et al., 1998). In the present study, roots grown in heterotrophic condition displayed a very high CWIN activity (Figure 1A). In heterotrophic cell suspension culture of maize, metabolizable sugars, sucrose and glucose, were associated with the increased abundance of *Incw1* transcripts, the concomitant increased levels of INCW1 protein and enzyme activity (Cheng et al., 1999). Taken together, these data indicate that assimilation of external sugars *via* the increased activity of apoplastic invertases may help to compensate the reduced input of photoassimilates in roots. Considering that sucrose is known to act as signaling molecule (Rolland et al., 2002), it also indicates that sucrose availability in the medium probably modulates the level of CWIN activity by up regulating *CWIN* gene expression (Koch, 2004).

Numerous studies have reported an increase in CWIN activity upon pathogen challenges with bacteria, fungi, virus, oomycetes or nematodes (Proels and Hückelhoven, 2014; Tauzin and Giardina, 2014). In many pathosystems, CWIN induction is frequently accompanied by a reduction of the photosynthesis, an accumulation of soluble carbohydrates, and a reduction in carbon export from infected leaves, leading to an enhanced sugar flow toward the infection site (Scharte et al., 2005; Berger et al., 2007). Together, these changes in carbon partitioning suggest that pathogen infections lead to the establishment of a new sink competing with existing sinks (Schultz et al., 2013). The involvement of invertases during the interaction of plants with biotrophic pathogens is particularly well documented (Tauzin and Giardina, 2014). For example, the induction of *AtCWIN1* was correlated with an increased CWIN activity upon infection with the biotrophic fungus *E. cichoracearum* (Fotopoulos et al., 2003). In grape, the cell wall invertase gene *VvcwINV* is also up regulated upon infection with downy and powdery mildews (Hayes et al., 2010). To date, few studies analyzed the role of CWIN during the interaction between plants and pathogens with a necrotrophic lifestyle. To address the specific case of necrotrophs, we challenged mature leaves with the fungus *B. cinerea*. *Arabidopsis* leaves inoculated with *B. cinerea* showed an increased CWIN activity during the course of the infection (Figure 4A). Ruiz and Ruffner (2002) reported such an induction of CWIN activity in infected grape berries and evidenced the contribution of fungal invertases. Interestingly, the absolute level of the *Botrytis*-induced CWIN activity in *Arabidopsis* leaves reached the one of roots (Figures 1A, 4A). Furthermore, this induction occurred at late stage, when the interaction with the fungus is well established. Altogether, our results suggest that leaf infection with the necrotrophic fungus *B. cinerea* may lead to a source/sink transition with the creation of a new sink. The question whether infected leaves may compete with other sinks for resources remains to be determined.

### Transcriptional and post-translational controls of CWIN activity

To identify molecular actors that may contribute to CWIN activity, we have conducted a transcriptional approach. Cell wall invertases, which belong to the GH32 hydrolases family, are encoded by a small multigenic subfamily. Transcriptional studies by RT-qPCR revealed that only two *AtCWIN* genes, *AtCWIN1* and -*5*, were expressed in leaves and roots (Figure 1B). According to Ruan (2014), *AtCWIN*-encoding genes form 2 groups, with a constitutive expression in all tissues for *AtCWIN1* and *AtCWIN5*, and a predominant expression in reproductive organs for *AtCWIN2* and *AtCWIN4*. Our results are in agreement with several reports indicating that *ACWIN1* was highly expressed in source leaves (Sherson et al., 2003; Quilliam et al., 2006). In our experimental conditions, *AtCWIN5* transcripts were barely detected which was different from the report made by Quilliam et al. (2006). Although the role of CWIN in sinks is well established, the expression pattern of *AtCWINs* genes in roots was not clearly defined. In this study, we have shown that both *AtCWIN1* and -*5* were expressed in soil-grown roots (Figure 1B). However, *AtCWIN1* transcripts were largely predominant in roots cultured in heterotrophic condition. This latter result, together with the high CWIN activity, supports our hypothesis that CWIN activity is transcriptionally controlled by the sucrose abundance (Cheng et al., 1999). After challenge with *B. cinerea*, solely *AtCWIN1* was up regulated (Figure 4B). It seems that transcriptional activation of this particular gene is part of a general response from the plant to environmental stimuli, since it has been shown that *AtCWIN1* expression is stimulated under several biotic stresses (Chou et al., 2000; Fotopoulos et al., 2003; Bonfig et al., 2006; Siemens et al., 2011) and under abiotic stresses (Quilliam et al., 2006).

Many reports indicate that CWIN activity also depends on posttranslational regulation (Rausch and Greiner, 2004). To address this question, we monitored the expression of *AtCIF1* and *AtC/VIF2* genes encoding specific inhibitor proteins (Link et al., 2004; Su et al., 2016). Our results suggest that CWIN activity is maintained at a low level in healthy source leaves by a mechanism that likely involves the posttranslational activity of the invertase inhibitor AtC/VIF2. Upon *Botrytis* challenge, the transcriptional repression of *AtC/VIF2* is accompanied with the induction of *AtCWIN1* gene expression (Figures 4B,C). As a consequence, the CWIN-mediated increase of sucrose cleavage is promoted in infected leaves. A similar mechanism of posttranslational control of CWIN activity through the repression of *AtC/VIF2* has been reported in response to infection by *P. syringae* (Bonfig et al., 2010).

### *AtCWIN1* is essential for CWIN activity

We analyzed the T-DNA insertion line *cwin1-1* that displays an impaired expression of *AtCWIN1* (Supplementary Figure 2B). In *cwin1-1*, leaf CWIN activity was completely abolished, whereas only a weak residual activity was detected in roots (Figure 1A), demonstrating the essential contribution of *AtCWIN1* in apoplastic sucrose cleaving activity. In a previous study, Quilliam et al. (2006) reported that the insertional inactivation of *AtCWIN1* led to only a 30–50% reduction in leaf CWIN activity. This apparent discrepancy may be due to the use of a different T-DNA insertion line (SAIL_258_A01). In the present work, we showed that *AtCWIN1* is the only *AtCWIN* gene induced under *Botrytis* attack and its inactivation leads to the complete loss of the *Botrytis*-induced increase in CWIN activity (Figure 4A), demonstrating its unique role upon infection. A similar loss of the wound induction of CWIN activity has been reported in Δ*atcwin1* plants (Quilliam et al., 2006). Since responses to wounding and pathogens share overlapping signaling pathways (Fujita et al., 2006), it reinforces the hypothesis that CWINs regulate common stress responses of the plant (Roitsch et al., 2003).

### Different routes for sucrose assimilation

**Figure 9:**
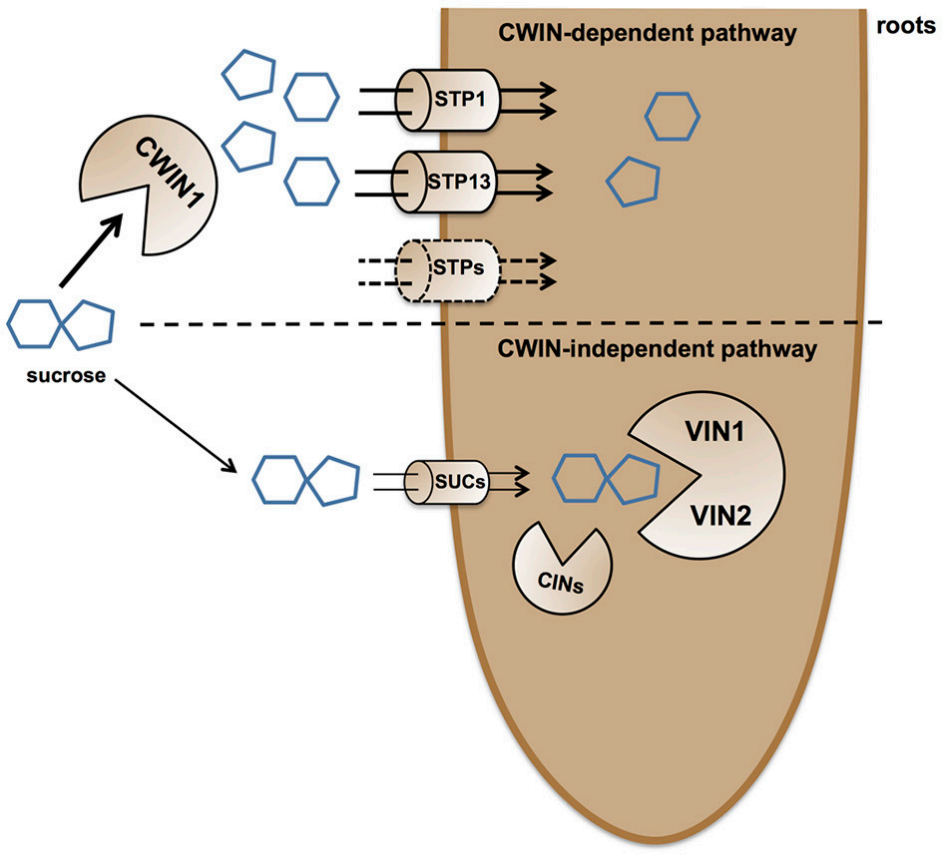
**Simplified model of external sucrose retrieval from the surrounding environment**. At the root/environment interface, sucrose can be absorbed through two independent pathways. The main route for sucrose assimilation is driven by a *AtCWIN1*-dependent pathway, resulting in sucrose hydrolysis into glucose and fructose. Hexoses are then taken up by the coordinated activity of hexose specific transporters, such as AtSTP1 and AtSTP13. The alternative CWIN-independent pathway relies on sucrose transporters (most likely AtSUCs) for sucrose internalization and the involvement of intracellular invertases (VINs and CINs) for the generation of free hexoses to support the plant primary metabolism. The vacuole is not shown.

Because *cwin1-1* plants exhibited a dramatically low rate of apoplastic sucrose cleavage, it provides an ideal tool to explore the regulatory role of CWIN activity in the assimilation of carbohydrate by sinks. Using 9-day-old roots cultured *in vitro* and radiolabeled sucrose uptake experiments, we were able to propose a model for sucrose retrieval from the surrounding environment (depicted in Figure 9). First, we showed that roots from *cwin1-1* seedlings have reduced capacity to actively take up external sucrose (Figure 2B), indicating that this process is mainly dependent on the sucrolytic activity of *AtCWIN1*. Using T-DNA and CRISPR/Cas9 mutants impaired in hexose transport, we demonstrated that external sucrose is absorbed in the form of hexoses by a sugar/H^+^ symport system (Figure 2B). It involves several Sugar Transporter Proteins (STP) of the plasma membrane, i.e., STP1 and STP13, which are transcriptionally co-expressed with *AtCWIN1* in roots. This is supported by the work of Yamada et al. (2011) showing that monosaccharide absorption by roots depends on the expression of *STP1* and -*13*. In agreement with our data, a rapid and efficient mechanism of sucrolysis before import has been reported in root tips using FRET glucose and sucrose nanosensors (Chaudhuri et al., 2008). Because plants overexpressing hexose-specific transporters exhibited an increased sucrose uptake rate (Figure 2B), we further showed that the *AtCWIN1*-dependent conversion of sucrose into glucose and fructose is a non-limiting step to supply hexose transporters with substrates.

**Figure 10:**
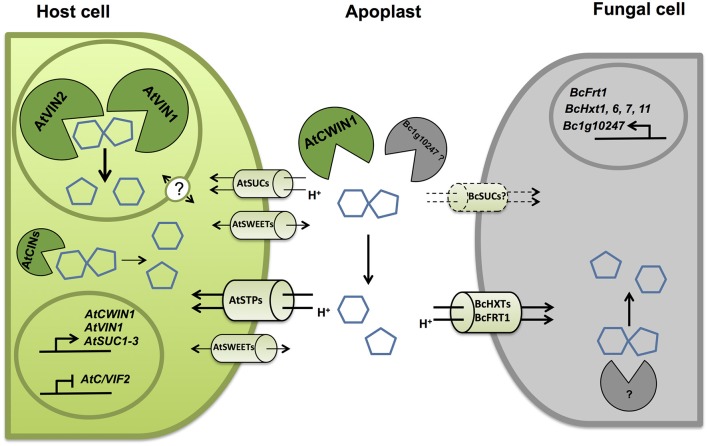
**Simplified model representing key molecular actors involved in the competition for sugars at the *A. thaliana*/*B. cinerea* interface**. Upon infection by *B. cinerea*, unloaded sucrose is mainly cleaved into glucose and fructose. Cell wall invertases from host (*AtCWIN1*) contribute to the accumulation of hexoses in the apoplast through transcriptional and posttranslational regulations. Host cells can assimilate free hexoses *via* the induced activity of hexose-specific transporters belonging to the Sugar Transporter Protein (STPs) family. *B. cinerea* possesses its own functional sucrolytic machinery (Bc1g10247 ?) and also a multigenic hexose uptake system (BcHXTs and BcFRT1). A secondary pathway for apoplastic sucrose retrieval involving sucrose transporter has been evidenced. Intracellular sucrolytic machinery, involving VINs and to lesser extent CINs, is efficient to provide intracellular hexoses to maintain sugar homeostasis in host cells and to fuel plant defenses. The precise regulatory role of tonoplastic, plasma membrane sucrose transporters and sugar facilitators (SWEETs) needs to be elucidated.

Besides this *AtCWIN1*-dependent pathway, this study has also brought to light the involvement of a second route for the retrieval of external sucrose (Figure 9), since a substantial proportion of sucrose was imported into *cwin1-1* seedling roots (Figure 2B). This *AtCWIN1*-independent pathway likely involves an active sucrose transport system, because sucrose uptake rate was almost totally abolished in *cwin1-1* roots in presence of the protonophore CCCP (Figure 2A). This result is different from the study using FRET glucose and sucrose sensors showing that sucrose accumulation in root tips was insensitive to protonophores, suggesting the involvement of low-affinity transporters for root tip uptake (Chaudhuri et al., 2008). As we used completely different experimental conditions (e.g., entire root system vs. root tip, radiolabeled sucrose vs. FRET sensor, sugar and CCCP concentrations), it is likely that both studies highlighted complementary processes. However, according to several reports, we found that three sucrose transporters *AtSUC1*, -*2*, and -*3* were expressed at a high level in roots supporting the probable involvement of sucrose/H^+^ symporters in root sucrose retrieval (Figure 2E) (Truernit and Sauer, 1995; Meyer et al., 2004; Sivitz et al., 2007, 2008; Durand et al., 2016). Therefore, it would be interesting to determine the precise role of individual *AtSUC* genes in sucrose uptake in future studies. The *AtCWIN1*-independent pathway can also operate upon infection with *B. cinerea* (model in Figure 10). It was technically impossible to measure sucrose uptake in infected leaves because leaf tissues were too damaged. However, results showing that 3 *AtSUC* genes were expressed in both WT and *cwin1-1* infected leaves, two of them being slightly induced (Figure 8), help to sustain this hypothesis.

### Impact of the CWIN deficiency in growth and pathogen resistance

The inactivation of *AtCWIN1* did not cause any obvious alteration of phenotype despite an almost abolished CWIN activity in both source leaves and roots. Similar observation has been reported by Quilliam et al. (2006). It suggests that the *AtCWIN1*-mediated activity is not essential for *A. thaliana* growth and development. Previous studies have reported contrasting results concerning the impact of CWIN activity on plant growth and development. For example, antisense repression of the carrot *DcCWIN1* gene strongly modified carbon partitioning, leading to the alteration of development, inhibition of tap root formation and enhanced foliar growth (Tang et al., 1999).

We evidenced that *AtCWIN1* is unique among *CWIN* genes regarding its essential contribution to the CWIN activity during *A. thaliana*/*B. cinerea* interaction. However, phenotypic analysis revealed that lack of *AtCWIN1* did not cause any modification of the basal resistance against *B. cinerea* (Figure 5). By contrast, several reports indicate that induction of CWIN activity may be required for plant resistance to pathogens (Bonfig et al., 2010; Siemens et al., 2011). The differential impact of CWIN activity in the susceptibility or resistance may be due to the difference of the pathogen lifestyle. The degradation of apoplastic sucrose by CWIN is crucial for biotrophic or hemibiotrophic pathogens as they mainly rely on the sugar acquisition from the apoplast through the activity of hexose transporters (Sutton et al., 1999; Hall and Williams, 2000; Voegele et al., 2001). As a necrotroph, *B. cinerea* secretes multiple cell wall-degrading enzymes that allow plant tissue colonization and the release of carbohydrates for consumption (Choquer et al., 2007). Consequently, CWIN activity may be of secondary concern for pathogen development in the case of necrotrophs. In some reports, it was also shown that during infection, CWIN activity triggers plant defense responses (Essmann et al., 2008b; Tauzin and Giardina, 2014). Because we did not observe any modification of classical defense gene expression patterns (Supplementary Figure 5), we postulate that *AtCWIN1* is not essential for the activation of the PAMP-Triggered Immunity in response to *B. cinerea*. However, the precise role of free hexoses generated by the *Botrytis*-induced activity remains to be elucidated.

### Alternative sucrose cleavage by intracellular invertases

Considering the importance of cell wall invertases in plant carbon partitioning, it was quite unexpected that *cwin1-1* plants grew up normally, either in soil or *in vitro*. We attempted to understand how plants deal with the absence of CWIN activity. Interestingly, we found that the content of the most important soluble sugars were similar in WT and *cwin1-1* leaves and roots (Supplementary Figure 4). These results strongly suggest that *cwin1-1* tissues are sufficiently fuelled with hexoses by an alternative sucrolytic activity. In addition to CWINs, cytosolic (CIN) and vacuolar (VINs) invertases are important in maintaining the intracellular sugar homeostasis. Roles of CINs are less clear, probably because they are less stable proteins with low activity compared to VINs and CWINs (Pagny et al., 2003; Roitsch and González, 2004; Ruan, 2014). Therefore, we focused our analysis on vacuolar invertases known to play an essential role for normal growth (Sergeeva et al., 2006; Ruan et al., 2010; Leskow et al., 2016). Consistent with other studies, we were able to measure a transcript accumulation of VIN-encoding genes and high VIN activities in leaves and roots (Figure 3) (Quilliam et al., 2006; Sergeeva et al., 2006; Wang et al., 2010; Leskow et al., 2016; Su et al., 2016). VIN activity was operational in *cwin1-1* leaves and roots, and a comparative analysis of absolute levels of sucrose degradation showed that the invertase activity was far greater in the vacuole that in the apoplast (Figures 1A, 3B).

We monitored cytoplasmic and vacuolar invertase activities in infected leaves. Interestingly, the overall intracellular invertase activity was induced in WT and *cwin1-1* infected leaves, with a major contribution of VINs (Figures 6A–C). The involvement of vacuolar and cytosolic invertases in plant defense is poorly described. Sutton et al. (2007) reported that the infection of wheat leaves with powdery mildew (*Blumeria graminis*) induced all types of invertases. The down-regulation of a wheat alkaline/neutral invertase correlates with reduced host susceptibility to wheat stripe rust caused by *Puccinia striiformis* (Liu et al., 2015). Altogether, our results highlight the importance of the VIN-mediated sucrose degradation. In the absence of *AtCWIN1*, we postulate that sucrose might follow an alternative pathway involving sucrose transporters and VINs (Figures 9, 10). We hypothesize that the high activity of the latter pathway is sufficient to maintain the internal sugar homeostasis and may provide sufficient hexoses to fuel plant defense. In this scenario, the precise role of tonoplast sugar transporters involved in balancing cytosolic and vacuolar sugar levels has to be determined (Schulz et al., 2011; Hedrich et al., 2015).

### Competition for sugars at the plant/pathogen interface

The impact of the increased CWIN activity for both the host and the pathogen remains to be elucidated. On one side, it may be advantageous for the host by providing resources to defending cells (Biemelt and Sonnewald, 2006; Schultz et al., 2013). This assumption required the cooperation of CWIN and hexose transporters in order to supply host tissues with hexoses. During the biotrophic interaction between *E. cichoracearum* and *A. thaliana*, the up-regulation of *AtCWIN1* and the hexose transporter *AtSTP4* is correlated with the increase in invertase activity and glucose assimilation (Fotopoulos et al., 2003). In a previous study, we have reported that the high expression of the hexose transporter *STP13* is associated with an increased glucose uptake activity, leading to an enhanced resistance against *B. cinerea* (Lemonnier et al., 2014). Since STP13 is the only member of the AtSTP family induced by the necrotrophic fungus *B. cinerea* (Lemonnier et al., 2014), it may have a coordinated role with *AtCWIN1* to efficiently supply host cells with hexoses.

Increased CWIN activity may also be beneficial for the nutrient acquisition of the fungus since it may reduce the sucrose exported from leaves, facilitate local phloem unloading and increase the content of available apoplastic hexoses (Scharte et al., 2005). To gain insight into the strategy of carbon assimilation by *B. cinerea*, we explored the capacity of this fungus to cleave sucrose and absorb external sugars. Although all types of invertase activities were detected, our results indicate that sucrose is preferentially cleaved into hexoses prior to be internalized by an active hexose uptake system (Figures 8, 10). To date, few studies have reported the characterization of fungal CWINs (Voegele et al., 2006). Regarding necrotrophs, fungal specific invertases have been detected in infected grape and sunflower infected with *B. cinerea* and *Sclerotinia sclerotiorum*, respectively (Ruiz and Ruffner, 2002; Jobic et al., 2007). In order to identify candidate genes encoding putative invertases, we have targeted genes belonging to the glycoside hydrolase family 32 in the genome of *B. cinerea*. Based on conserved motifs and expression pattern during plant infection (Figure 8D), we have identified a candidate with a putative function of cell wall invertase. The next challenge will be to characterize its activity *in vitro* and *in vivo*, and to determine its role in the sugar acquisition and fungal pathogenicity. *Botrytis* sucrose transporters have not been yet identified but a versatile hexose uptake system, including a high affinity fructose transporter (BcFRT1) and a multigenic family of putative hexose transporters (BcHXTs) have been described (Doehlemann et al., 2005; Dulermo et al., 2009). Our results showing that sugars are preferentially assimilated in the form of hexoses by the fungus are supported by the differential expression of several hexose transporters during the course of the pathogenic interaction (Figure 10).

By targeting the *AtCWIN1* gene, the present study had for main purpose to explore the role of cell wall invertase activity in source and sinks organs, and upon pathogen infection using the necrotrophic fungus *B. cinerea*. We conclude that *AtCWIN1* is essential for the cell wall invertase activity in diverse situations, i.e., in sink organs or in infected leaves. However, our results show that plant cells have evolved alternative mechanisms for sucrose cleavage to maintain the cellular homeostasis (Figures 9, 10). The next challenge will be to determine the precise role and regulation of the molecular actors involved in these alternative pathways. Further investigations are needed to understand more precisely the role of cell wall invertases in the competition for sugars at the plant/pathogen interface. One interesting track would be the assessment of fungal invertase and sugar transporter contributions in *B. cinerea* pathogenicity, as the fungus side in plant/pathogen interaction has retained much less attention than the plant side so far.

## Author contributions

SL, PC, and FV designed the experiments. FV and CG performed the experiments. SL and FV wrote the article. SL, PC, CG, and FV discussed the data and revised the article. All authors approved the final manuscript.

## Funding

This work was supported by the Centre National de la Recherche Scientifique, the University of Poitiers, the Région Poitou-Charentes (FV PhD grant), the State-Region Planning Contracts (CPER) and the European Regional Development Fund (FEDER).

### Conflict of interest statement

The authors declare that the research was conducted in the absence of any commercial or financial relationships that could be construed as a potential conflict of interest.
