# Supplementary material for: Targeting the AtCWIN1 Gene to Explore the Role of Invertases in Sucrose Transport in Roots and during Botrytis cinerea Infection
Source: Front Plant Sci. 2016 Dec 20;7:1899. doi: 10.3389/fpls.2016.01899 (PMC5167757; doi:10.3389/fpls.2016.01899)
Supplement: Supplementary file 1 [file Table1.PDF]

Supplementary table 1 : Oligonucleotides used in this study for cloning and selection of OESTP1 and *CR*-*stp1* lines.

Primer name	Target locus	Sequences	Observations
***AtSTP1* CDS F**	*AtSTP1*	CACCATGCCTGCCGGTGGAT	*AtSTP1* CDS cloning
***AtSTP1* CDS R**	*AtSTP1*	TCAAACATGCTTCGTTCCAG	
**Spacer-STP1 F**	*AtSTP1*	**ATTG**CGCAGTCGATAGAGCACCCG	sgRNA cloning
**Spacer-STP1 R**	*AtSTP1*	**AAAC**CGGGTGCTCTATCGACTGCG	
**SS42 (Fauser *et al*, 2014)**		TCCCAGGATTAGAATGATTAGG	Cloning control
**SS61 (Fauser *et al*, 2014)**		GAGCTCCAGGCCTCCCAGCTTTCG	
**SS43 (Fauser *et al*, 2014)**		CGACTAAGGGTTTCTTATATGC	
**HRM-*STP1* F**	*AtSTP1*	TTGGTCGCCGCTAGTAAAGA	HRM genotyping
**HRM-*STP1* R**	*AtSTP1*	GCTGTTGAAAGAACGGAATCA	
**sSTP1 F**	*AtSTP1*	TCCTCCCTGACACTCCCAAT	SANGER sequencing
**sSTP1 R**	*AtSTP1*	AAAACCCGCCACATAGATGC	
**OFF-T1 F**	*At4g13580*	CTGCTCTTTTTACTGCAACTACCG	Off-target assessment by HRM
**OFF-T1 R**	*At4g13580*	TCGTGCATGTACAATTCAAAAATC	
**OFF-T2 F**	*At4g07730*	CAGGACTTCTCTACAGGACTTCCA	
**OFF-T2 R**	*At4g07730*	CAGCTCTGGATCATGGCAGATA	
**OFF-T3 F**	*At5g31980*	GCATCGACCTCTACTCCCAAA	
**OFF-T3 R**	*At5g31980*	GACAGATGGTGATAGCGTGAGC	
**OFF-T4 F**	*At3g43430*	AAAGAAAGCCTAGCCGTGTCC	
**OFF-T4 R**	*At3g43430*	TCCTAGACACACGGCACACA	
**OFF-T5 F**	*At3g56600*	GGTGCCATTCACCGACATT	
**OFF-T5 R**	*At3g56600*	GCAGCAGCAGCTTCTTTCAA	
**Cas9 F**		AGTGCTCGATGCTACCCTCA	T-DNA detection
**Cas9 R**		GATCAGCCCTTGAATCACCA	
