# Supplementary material for: Targeting the AtCWIN1 Gene to Explore the Role of Invertases in Sucrose Transport in Roots and during Botrytis cinerea Infection
Source: Front Plant Sci. 2016 Dec 20;7:1899. doi: 10.3389/fpls.2016.01899 (PMC5167757; doi:10.3389/fpls.2016.01899)
Supplement: Supplementary file 2 [file Table2.PDF]

Supplementary table 2 : Primers used in this study for gene expression and DNA quantification analyzes.

Genes	Loci	Forward primers (F)	Reverse primers (R)
Reference (REF)	*At4g26410*	GAGCTGAAGTGGCTTCCATGAC	GGTCCGACATACCCATGATCC
AtACTIN2	*At3g18780*	CTTGCACCAAGCAGCATGAA	CCGATCCAGACACTGTACTTCCTT
AtSTP1	*At1g11260*	ATGCCGTCTTTCCTCAAGCG	TAGCGTCGGGCTATCGTACT
AtSTP2	*At1g07340*	GCTGTGTCATTGCTGCTGTT	TCGTACACGTGTGGGAAAAA
AtSTP3	*At5g61520*	GTAACTTGCGACACGAGCATCT	ACCGTTGGGTTTGGAGAGAGT
AtSTP4	*At3g19930*	CTTCTCTCTTTGCCTCCACCAT	CGACTCCGAAACCAAGTAGGA
AtSTP5	*At1g34580*	GGGGACGGAGAGATGAAGAAG	CTTGAAATCGCAGAGCGTTG
AtSTP6	*At3g05960*	GCACTCGTGGCCAGTTTCGTC	TGAGACCGACACCAATCAAG
AtSTP7	*At4g02050*	ACTTAGCTATGCTTCTTGCC	ATGAGTTGGTGCCACTTCTG
AtSTP8	*At5g26250*	GGCTAGCTTCTTTGCTTCGGCT	AGATGGAAGCGAGTTGCATT
AtSTP9	*At1g50310*	TGTGAGGCTGCTAAGAAAGTGG	TAAAGAGAACGGGGGCGTAGA
AtSTP10	*At3g19940*	CGGTAGACTGTTGCTTGGTGTT	GCTCCTCTTATCTTCGCTGGA
AtSTP11	*At5g23270*	CTTTCCTTGCCTCCACCATTAC	AATCCGACACCTACACCGAGA
AtSTP12	*At4g21480*	GACAATGGATTCGTTTCAGC	GAGAAACGCTGTCAAATCTGC
AtSTP13	*At5g26340*	TATGGGACCGCCAAGATTAAA	AAGCTCCGACCGTTAGAAGAA
AtSTP14	*At1g77210*	ACTCATGTTCCTCGGTGGAC	ACTTTGATCAGCACGGCTTT
AtSUC1	*At1g71880*	GACCTTTCGACGCCTTGTTC	AATACTCCACTAATCGCCGCTG
AtSUC2	*At1g22710*	GGTAAGTGGTGTATTGGCGTTG	GAGCCAAACAACCACTGCTAAA
AtSUC3	*At2g02860*	CAAGAACCGCAGCCGTAATC	CTTGACCGCCACCGGAAT
AtSUC4	*At1g09960*	AGTGTCAAGCGAGGAACGCATA	AGTCACACGAGAAGCCATTGC
AtSUC5	*At1g71890*	GGGCTATGGGATTCCATTAG	TAAAAGACAGACGACCAAGG
AtSUC6	*At5g43610*	TCCTGTCTCCGGCCTGCTT	AGGCGCCCATAGCGATGA
AtSUC7	*At1g66570*	GTCTTTAAGAGACAAGCCCAC	AGACTGTCTATCCACAGTCGT
AtSUC8	*At2g14670*	CTAGCTTCCATAATCTCAAGT	TTGGTAAGTTTCCACCTCCAAAA
AtSUC9	*At5g06170*	GTGGTTCCTGATGAGCCG	GAGAAGCTGAACGTATGGG
AtCWIN1	*At3g13790*	CGGAAGTGGAATGTGGGAAT	TGAAACCATTGTCCGGTACG
AtCWIN2	*At3g52600*	ACATGGTCCGGTTCAGCTAC	CTTGAGGTATGGGTCGGAAA
AtCWIN4	*At2g36190*	CAATCGTACCGGGAAAAGGA	CCGTCTTTGGAGAACCAAGC
AtCWIN5	*At3g13784*	GGGTCGGTAATGGAGGTTCA	AGCCCGGTTCAATCACATCT
AtVIN1	*At1g62660*	CGGCGTCGTTTACGGTCT	TCATCAGGTGCTGGAAGGAA
AtVIN2	*At1g12240*	GCATTCGGACAAGGTGGAAG	TAACCGTCGCATCAAGAGCA
AtCIF1	*At1g47960*	ACACTTCTGGCCTCGCTCTC	AATGGCTTCGGGAACATCAG
AtC/VIF2	*At5g64620*	TCTCATCTTCCTCCTCCTCGTT	ACCTTTTGTGTCGGCTGTGG
AtAtPDF1.2	*At5g44420*	TTTGCTGCTTTCGACGCAC	CGCAAACCCCTGACCATG
AtPAD3	*At3g26830*	TGCTCCCAAGACAGACAATG	GTTTTGGATCACGACCCATC
AtPR1	*At2g14610*	AAGGGTTCACAACCAGGCAC	CACTGCATGGGACCTACGC
AtiASK	*At5g26751*	CTTATCGGATTTCTCTATGTTTGGC	GAGCTCCTGTTTATTTAACTTGTACATACC
BcCutA	*Z69264*	AGCCTTATGTCCCTTCCCTTG	GAAGAGAAATGGAAAATGGTGAG
BcHxt1	*Bc1g09068*	CCACCATGGCTGGCTTCTA	TTCGGCTACCTCGGTCTCAA
BcHxt2	*Bc1g10092*	TCGGTACCGCTGTCCTCTTC	GGCTTCGTCTTCACGTCCAA
BcHxt3	*Bc1g11064*	GCCGTCGAAGGTGTTTACGA	CGACCCACCCTTGTGTAACAG
BcHxt4	*Bc1g14590*	CCACAAGATGGAAAAGGACCAA	GGCCCCGAAGAAAGCAA
BcHxt5	*Bc1g0533*	TCACCTTCCCCCTTATGTCAGT	CGGGTAGTTCCCTCTGGAATC
BcHxt6	*Bc1g03115*	AGGGTCTTTCCCTTGAGCAAGT	GAACCCATTTGGCGGAGTT
BcHxt7	*Bc1g10771*	CTGGCCGGCAATTTGG	TTTGTGGGAAAGTGAAAAGGAGTAG
BcHxt8	*Bc1g04637*	GAACTGCATCATCGGAGTCATC	AGCGCCGAGGTTTGCAT
BcHxt9	*Bc1g09522*	ACGTCGCGCGGTATTCAT	ACAGCCAAGGAGAGCATACACA
BcHxt10	*Bc1g09797*	TGGGTGCTCGTTGCATCTAG	GGATGATTTGGATGCTCAAAGG
BcHxt11	*Bc1g09891*	GGGTTGTCTGCGGAGAGATATT	GTGGCGACCGACATGGA
BcHxt12	*Bc1g10026*	TCGCCGGTCTCATTTTCC	TTTTCTGGATGTCCATGATCGA
BcHxt13	*Bc1g11623*	GCGGTCGGTCTGCTCAGTAT	ACTCAATCCCACAATCAATCCTCTA
BcHxt14	*Bc1g12037*	TGCGCCGCTCTTATGCTT	CTGGGATTGAAATGAGGAGGAT
BcHxt15	*Bc1g12561*	CCCCACTCGTCTCCGTTCT	GGAAAAATTGCACAACCATTGA
BcHxt16	*Bc1g13350*	CGGAGGTGCCATATCTGCTT	CGATCAGCATTCCAACATTGG
BcHxt17	*Bc1g13828*	GCCGCACATAAAGACACTCAAA	TGGAATAGCCATGCCCTCTAA
BcFrt1	*AY738713*	CCAGAATCCCCCCGTTTC	TTTCCAGACACCCCAAGCA
BcActA	*AJ000335*	CCGTGCTCCAGAAGCTTTGT	GTGGATACCACCGCTCTCAAG
BcTubA	*Bc1g05600*	GCGTTCGTGCATTGGTATGT	CACGGGCCTCAGAGAATTCA
	Bc1g10247	TCTCGCAAACGTCACACGTA	GGGGTTCGGATTCGTTGTAG
	Bc1g16010	CGGGTACGTCGCTTACAAAA	CCGTGAAGATCCGCAAGTAA
