# Supplementary figures and images for: Targeting the AtCWIN1 Gene to Explore the Role of Invertases in Sucrose Transport in Roots and during Botrytis cinerea Infection
Source: Front Plant Sci. 2016 Dec 20;7:1899. doi: 10.3389/fpls.2016.01899 (PMC5167757; doi:10.3389/fpls.2016.01899)

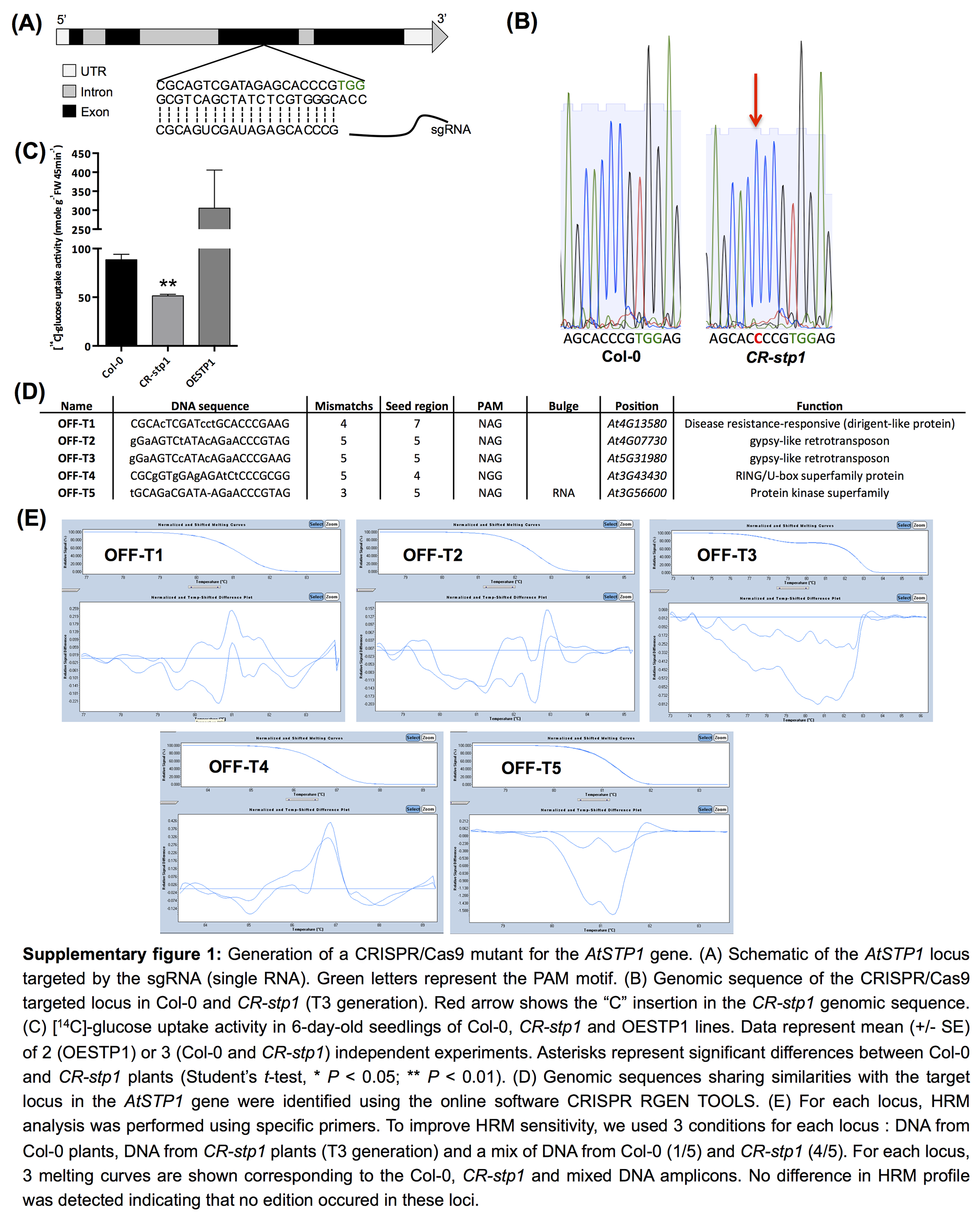

Supplement: Supplementary file 3 [file Image1.tiff]

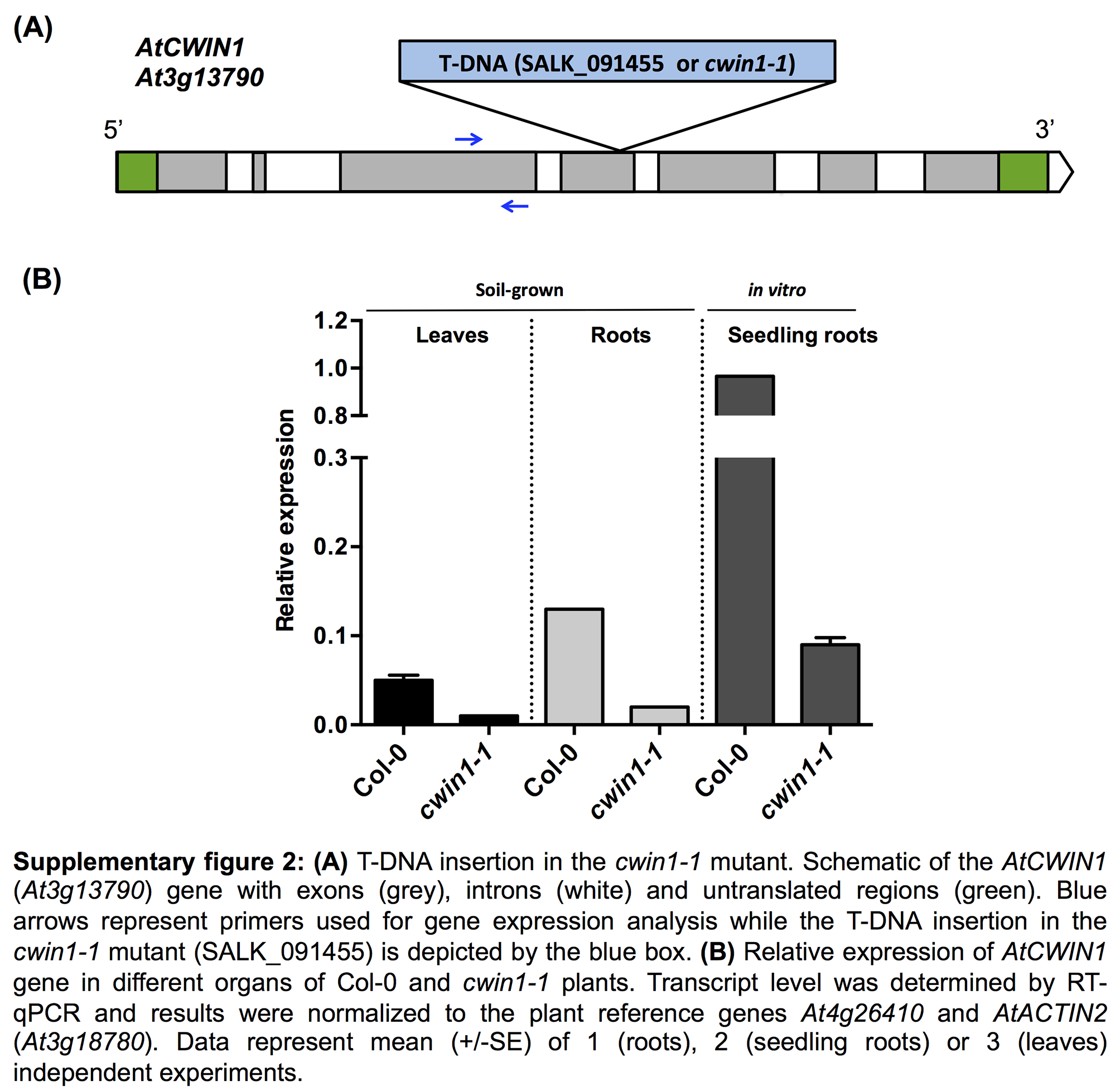

Supplement: Supplementary file 4 [file Image2.TIFF]

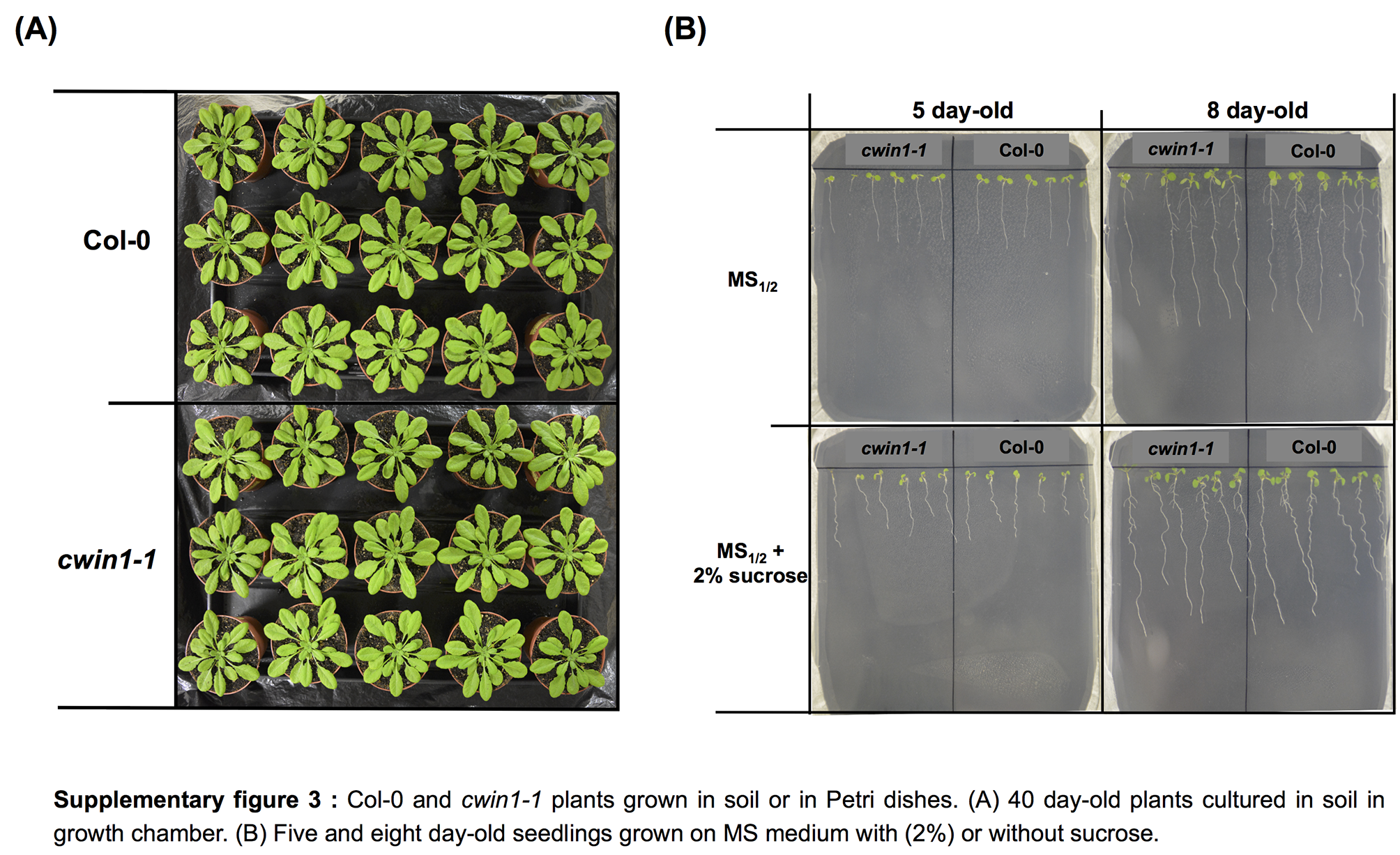

Supplement: Supplementary file 5 [file Image3.TIFF]

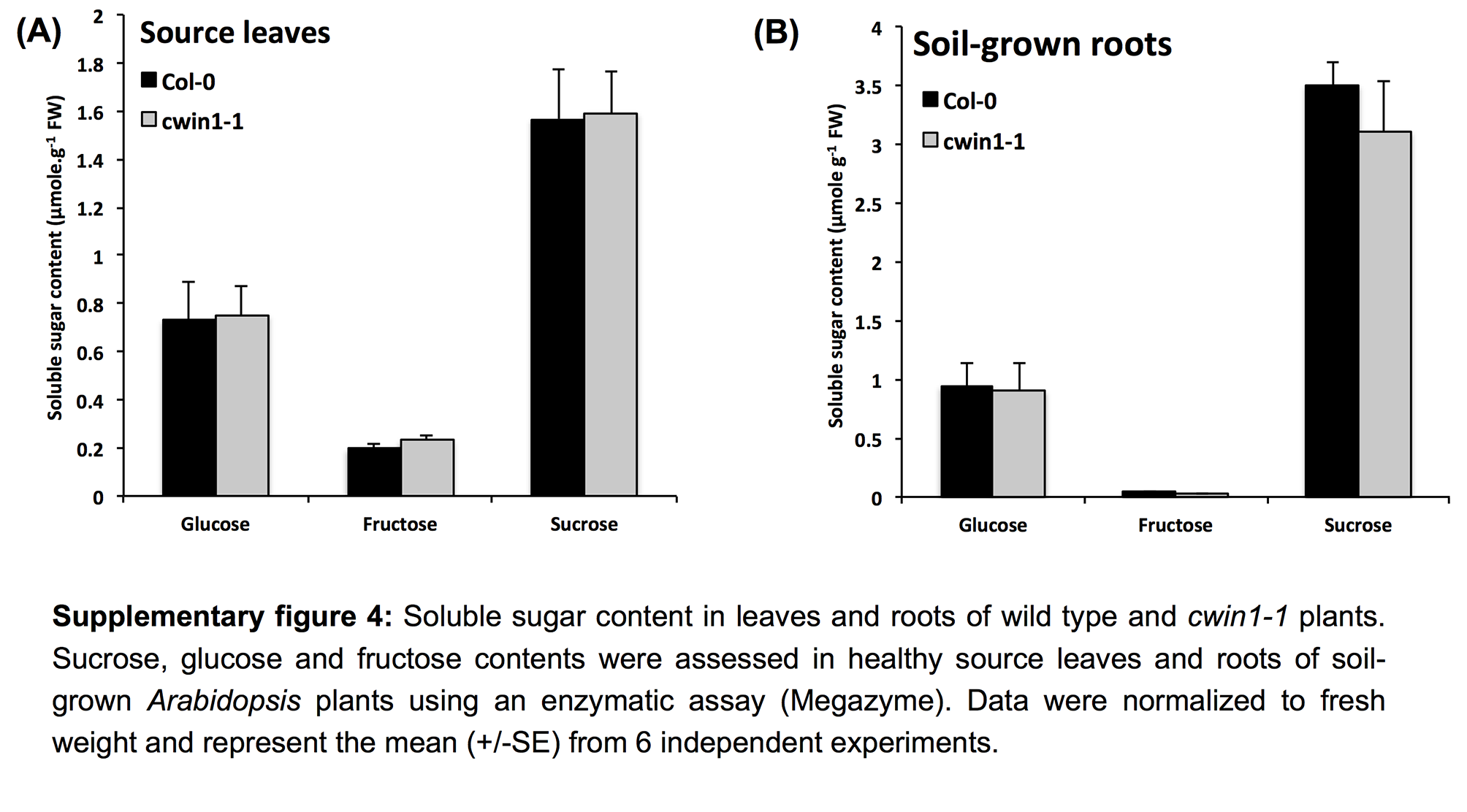

Supplement: Supplementary file 6 [file Image4.TIFF]

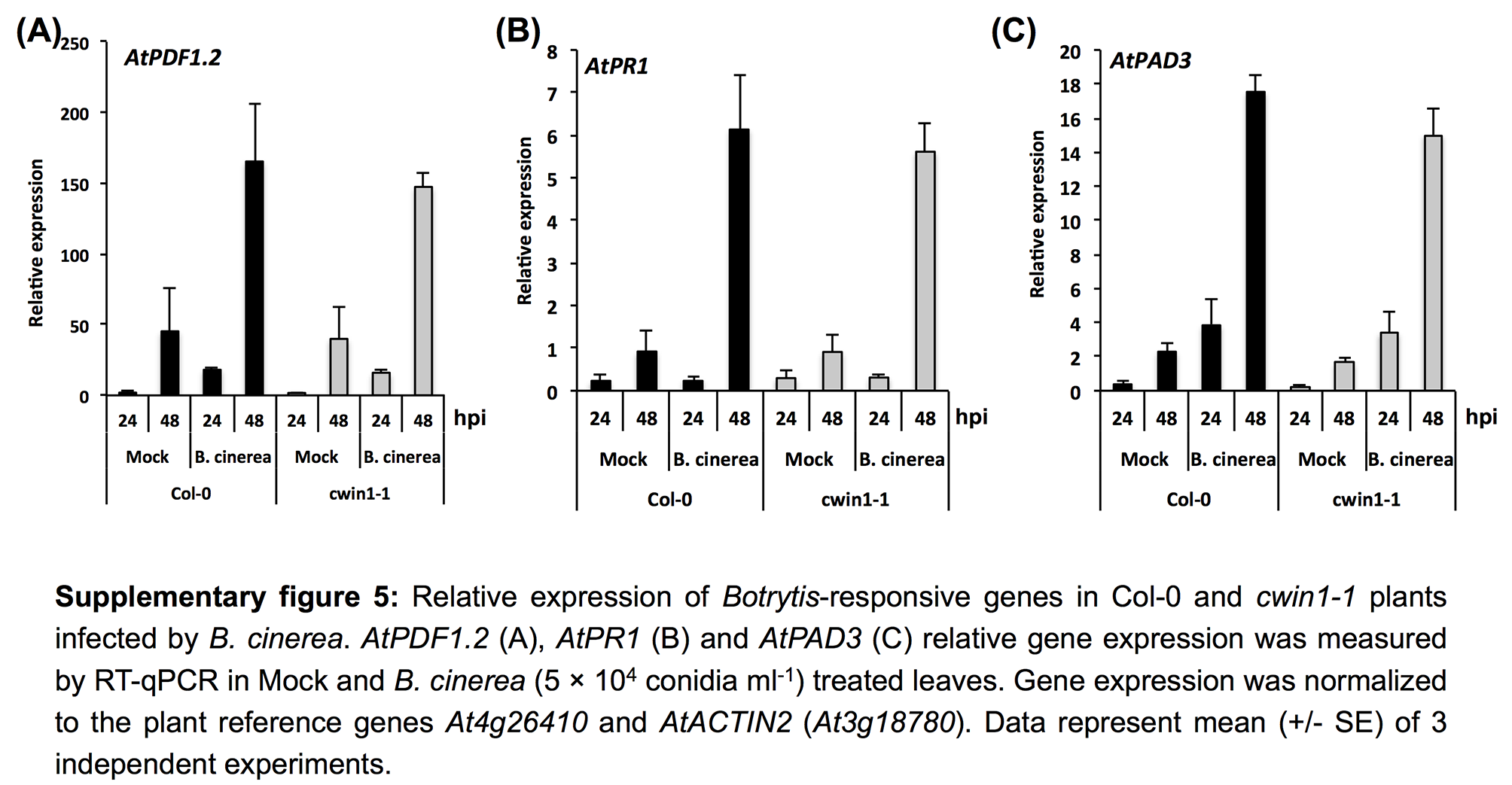

Supplement: Supplementary file 7 [file Image5.TIF]
